# Effect of Vitamin D_3_ Supplementation in the First 2 Years of Life on Psychiatric Symptoms at Ages 6 to 8 Years: A Randomized Clinical Trial

**DOI:** 10.1001/jamanetworkopen.2023.14319

**Published:** 2023-05-19

**Authors:** Samuel Sandboge, Katri Räikkönen, Marius Lahti-Pulkkinen, Helena Hauta-alus, Elisa Holmlund-Suila, Polina Girchenko, Eero Kajantie, Outi Mäkitie, Sture Andersson, Kati Heinonen

**Affiliations:** 1 Psychology/Welfare Sciences, Faculty of Social Sciences, University of Tampere, Tampere, Finland; 2 Population Health Unit, Finnish Institute for Health and Welfare, Helsinki, Finland; 3 Department of Psychology and Logopedics, Faculty of Medicine, University of Helsinki, Helsinki, Finland; 4 Queen's Medical Research Institute, University of Edinburgh, Edinburgh, United Kingdom; 5 Children’s Hospital, Pediatric Research Center, University of Helsinki and Helsinki University Hospital, Helsinki, Finland; 6 Research Unit for Pediatrics, Dermatology, Clinical Genetics, Obstetrics and Gynecology, Medical Research Center Oulu, Oulu University Hospital and University of Oulu, Oulu, Finland; 7 Research Program for Clinical and Molecular Metabolism, Faculty of Medicine, University of Helsinki, Helsinki, Finland; 8 Department of Clinical and Molecular Medicine, Norwegian University of Science and Technology, Trondheim, Norway; 9 Institute of Genetics, Folkhälsan Research Center, Helsinki, Finland; 10 Department of Molecular Medicine and Surgery, Karolinska Institutet, Stockholm, Sweden; 11 Department of Clinical Genetics, Karolinska University Hospital, Stockholm, Sweden; 12 Lawrence S. Bloomberg Faculty of Nursing, University of Toronto, Toronto, Canada

## Abstract

**Importance:**

Vitamin D is associated with neurodevelopment, but causality, critical windows, and potentials for modification remain unknown.

**Objective:**

To determine the impact of high-dose (1200 IU) vs standard-dose (400 IU) vitamin D_3_ supplementation during the first 2 years on psychiatric symptoms at ages 6 to 8 years and whether the impact is different in children with lower vs higher maternal vitamin D_3_ levels; lower vs higher levels were defined as 25-hydroxyvitamin D (25[OH]D) less than 30 ng/mL vs 30 ng/mL or greater.

**Design, Setting, and Participants:**

This study was a long-term follow-up of the double-blind randomized clinical trial (RCT) Vitamin D Intervention in Infants (VIDI) conducted at a single center in Helsinki, Finland, at 60 degrees north latitude. Recruitment for VIDI took place in 2013 to 2014. Follow-up data for secondary data analysis were collected 2020 to 2021. VIDI originally included 987 term-born infants; 546 of these individuals participated in the follow-up at ages 6 to 8 years, among whom 346 individuals had data on parent-reported psychiatric symptoms. Data were analyzed from June 2022 to March 2023.

**Interventions:**

There were 169 infants randomized to receive 400-IU and 177 infants randomized to receive 1200-IU oral vitamin D_3_ supplementation daily from ages 2 weeks to 24 months.

**Main Outcomes and Measures:**

Primary outcomes were internalizing, externalizing, and total problems scores, with clinically significant problems defined as *T* scores of 64 or greater in the Child Behavior Checklist questionnaire.

**Results:**

Among 346 participants (164 females [47.4%]; mean [SD] age, 7.1 [0.4] years), the vitamin D_3_ dose was 400 IU for 169 participants and 1200 IU for 177 participants. Clinically significant internalizing problems occurred in 10 participants in the 1200-IU group (5.6% prevalence) compared with 20 participants (11.8%) in the 400-IU group (odds ratio, 0.40; 95% CI, 0.17-0.94; *P* = .04) after adjustment for sex, birth season, maternal depressive symptoms at birth, and parental single status at follow-up. In a post hoc subgroup analysis, 48 children in the 400-IU group with maternal 25(OH)D concentrations less than 30 ng/mL had higher internalizing problems scores compared with children in the 1200-IU group, including 44 children with maternal 25(OH)D concentrations below 30 ng/mL (adjusted mean difference, 0.49; 95% CI, 0.09-0.89; *P* = .02) and 91 children with maternal concentrations above 30 ng/mL (adjusted mean difference, 0.37; 95% CI, 0.03-0.72; *P* = .04). Groups did not differ in externalizing or total problems.

**Conclusions and Relevance:**

This randomized clinical trial found that higher-than-standard vitamin D_3_ supplementation in the first 2 years decreased risk of internalizing problems at ages 6 to 8 years.

**Trial Registration:**

ClinicalTrials.gov Identifiers: NCT01723852 (VIDI) and NCT04302987 (VIDI2)

## Key Points

**Question:**

Does higher daily vitamin D_3_ supplementation up to age 2 years decrease the risk of psychiatric symptoms at ages 6 to 8 years compared with the standard recommended dose?

**Findings:**

In this secondary analysis of a randomized clinical trial including 346 children, those randomized to higher vitamin D_3_ supplementation were less likely to have clinically significant internalizing problems.

**Meaning:**

This study found that higher than standard vitamin D_3_ supplementation up to age 2 years decreased the risk for internalizing problems in later childhood.

## Teaser

This secondary analysis of a randomized clinical trial investigates the effect of increased vitamin D supplementation up to age 2 years on the risk of internalizing, externalizing, and total psychiatric problems at ages 6 to 8 years.

## Introduction

Vitamin D insufficiency and deficiency are estimated to occur among almost half and more than one-tenth of the global population, respectively, across all ages.^1,2^ In addition to its well-known role in skeletal health, vitamin D also plays a role in neurodevelopment. Receptors and metabolizing enzymes for vitamin D are present in various areas of the human brain,^3^ and experimental animal studies have linked vitamin D deficiency to abnormal brain development.^4^

Approximately one-eighth of children in high-income countries have mental disorders,^5^ and much is still unknown regarding their etiology. Results from previous studies, which were primarily observational,^6,7^ suggested that lower childhood vitamin D levels, measured as serum 25-hydroxyvitamin D (25[OH]D), were associated with autism spectrum disorder (ASD) and attention-deficit/hyperactivity disorder (ADHD). Lower childhood vitamin D levels have also been associated with increased levels of depressive symptoms^8^ and internalizing and externalizing problems^9^ later in childhood. Causality, however, can be verified only using randomized clinical trials (RCTs). Our 2021 publication of a part of the double-blind interventional RCT Vitamin D Intervention in Infants (VIDI) study^10^ did not show benefits of a higher-than-standard vitamin D_3_ supplementation (1200 IU) between ages 2 weeks and 2 years compared with standard recommended supplementation (400 IU) on internalizing, externalizing, or dysregulation problems; competencies; or developmental milestones up to age 2 years. Conversely, the study found a potential small negative impact of higher-than-standard supplementation on externalizing problems. Behavioral and psychiatric problems may not be fully manifested during the early years but may become more evident when environmental demands increase.^11^ For example, the estimated earliest peak age at onset for anxiety and fear–related disorders is 5.5 years.^11^ Accordingly, the primary aim of this follow-up study was to build on our previous study and extend the inquiry to childhood psychiatric symptoms at ages 6 to 8 years. This period is characterized by increased demand for self-regulating skills, important in mitigating potential internalizing and externalizing problems, combined with a still-developing prefrontal cortex.^12^

Lower 25(OH)D levels in pregnancy have been associated with unfavorable neurobehavioral and mental health outcomes in offspring, including negative affectivity in infancy,^13^ attention-deficit/hyperactivity disorder,^14,15^ autism spectrum disorder,^16^ and depression.^17^ Therefore, our secondary aim was to explore whether a higher-than-standard childhood vitamin D_3_ supplementation modified the potential impact of maternal 25(OH)D levels during pregnancy on child mental health outcomes.

## Methods

Participating children’s parents signed informed consent forms at recruitment and at the 6 to 8–year follow-up for this RCT secondary analysis. Children gave written consent to participate at the 6 to 8–year follow-up. The study was approved by the ethics committee at the Hospital District of Helsinki and Uusimaa and registered with ClinicalTrials.gov (NCT01723852 [VIDI] and NCT04302987 [VIDI2]); it follows the Consolidated Standards of Reporting Trials (CONSORT) reporting guideline.

### Study Design and Participants

The VIDI study (see trial protocol in Supplement 1) is a double-blind, interventional RCT described previously in detail.^18,19,20^ The study originally comprised 987 families (492 female and 495 male infants) recruited from the Kätilöopisto Maternity Hospital in Helsinki, Finland, at 60 degrees north latitude between January 1, 2013, and June 30, 2014. All infants had Northern European ancestry. Infants were randomized to receive oral vitamin D_3_ supplementation at 400 IU (10 μg; 495 individuals) or 1200 IU (30 μg; 492 individuals) from ages 2 weeks to 2 years. Of recruited families, 12 did not meet inclusion criteria (Figure). Randomization was performed in blocks of 50 infants by a pharmacist at Helsinki University Hospital without relation to the study.^18^ Supplements were prepared by Orion Pharmaceuticals, and both groups received 5 drops daily. Parents received information of group membership after the 2-year intervention concluded. Self-administered questionnaires were used to collect information regarding parental health, lifestyle, and demographics. Information on gestation, delivery, and child demographics was derived from hospital records. Maternal serum samples were collected during routine maternity clinic follow-up visits at 6 to 27 weeks of gestation (mean [SD] gestation time, 11.3 [1.9] weeks) and stored in the Finnish Maternity Cohort serum bank as organized by the Finnish Institute for Health and Welfare. Samples were used to analyze maternal 25(OH)D concentrations.

**Figure.**
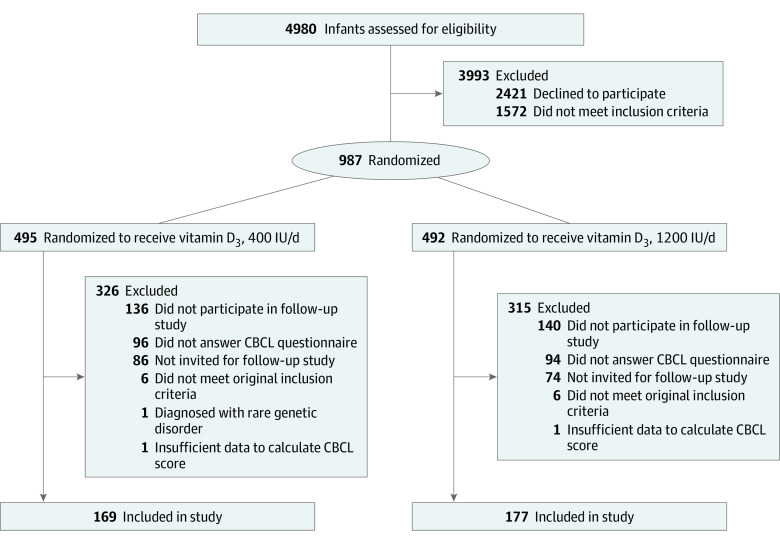
Flowchart of Study Enrollment, Allocation, and Follow-up CBCL indicates Childhood Behavior Checklist.

### Follow-up at 6 to 8 Years

The follow-up study was initiated in November 2019; we invited 817 families who remained in the original VIDI study until completion of the intervention at age 2 years of the child and had available home address info to participate. Of 546 families who participated in the follow-up study (55.3% of the original study population), 456 families completed online questionnaires regarding psychological and cognitive outcomes between September 2020 and May 2021. Personnel conducting follow-up were blinded to group membership. There was 1 participant excluded owing to diagnosis of a rare genetic disorder. The population of this long-term follow-up study consisted of 346 children whose parents completed the Child Behavior Checklist (CBCL) questionnaire (63.4% of those in the follow-up study); of the original 400-IU group, 169 children (34.6%) were included, and of the original 1200-IU group, 177 children (36.4%) were included. These children had somewhat more beneficial baseline characteristics regarding breastfeeding duration, maternal 25(OH)D level, smoking status, and parental education level compared with nonparticipants (eTable 1 in Supplement 2).

### Biochemical Analysis

Serum 25(OH)D concentrations were analyzed at the Pediatric Research Centre, University of Helsinki, using a fully automated IDS-iSYS immunoassay system with chemiluminescence detection (Immunodiagnostics System). Biochemical analyses are described in detail in the eAppendix in Supplement 2 and elsewhere.^18,19^ In this study, 30 ng/mL (to convert to nanomoles per liter, multiply by 2.496) was used as the cutoff point for maternal 25(OH)D concentration. This was previously suggested as a cutoff point for suboptimal 25(OH)D levels.^21^

### Outcome Measures

Childhood psychiatric symptoms were assessed at a mean (SD; range) age of 7.1 years (0.4; 6.3-8.2) years using CBCL, a standardized questionnaire comprising 113 items scored using a 3-point Likert scale (0 = absent; 1 = occurs sometimes; 2 = occurs often).^22^ Parent-reported questionnaires were used to calculate composite scores of internalizing, externalizing, and total problems (Cronbach α was 0.81, 0.89, and 0.93, respectively). Achenbach System of Empirically Based Assessment (ASEBA-PC) software version 3.0.136.0 (T.M. Achenbach) was used to convert raw scores to age and sex–standardized *T* scores; *T* scores of 64 or greater are considered to reflect clinically significant internalizing, externalizing, and total problems. We used *Z* scores of square root–transformed raw scores (0 = mean; 1 = 1 SD) and dichotomized *T* scores for internalizing, externalizing, and total scores as outcome measurements.

### Covariates

Potential covariates were assessed based on known association with childhood neurodevelopment or vitamin D_3_ levels. They were child age at CBCL assessment,^22^ child sex,^23^ maternal 25(OH)D level,^13,14,15,16,17^ birth season,^24,25^ gestational duration,^26^ maternal age at delivery,^27^ breastfeeding duration,^28^ parental educational level,^29^ parental single status at follow-up,^30^ maternal smoking status,^31^ and maternal depressive symptoms assessed at the maternity ward after childbirth^32^ (eAppendix in Supplement 2).

### Statistical Analysis

Longitudinal secondary data analyses followed the intention-to-treat principle; 2-tailed independent-sample *t* tests and Pearson χ^2^ tests were used to compare follow-up characteristics between intervention groups. Differences between groups in psychiatric problems were assessed using linear and logistic regression analysis. Results are presented without adjustment (model 1) and after adjustment for sex, maternal depressive symptoms at birth, birth season, and parental single status at follow-up (model 2). Model 2 covariates were associated with at least 1 psychiatric problems score (*P* < .05) or differed significantly in parental single status between supplementation groups. No other assessed potential covariates were associated with outcome variables or group membership and thus were not included in analyses (eTable 2 in Supplement 2). Comparing supplementation groups with α set at .05, we had a power of 0.80 to exclude or confirm effect sizes greater than 0.30 in mean difference (MD) for continuous variables and odds ratios (ORs) smaller than 0.42 in dichotomous variables. The potential impact of attrition bias was tested using inverse probability weighting estimation. In supplemental analyses, we tested associations of 1-year and 2-year 25(OH)D levels with psychiatric outcomes using regression analyses presented previously.

To test the potential interaction between prenatal 25(OH)D level and supplementation degree, children were grouped by maternal pregnancy 25(OH)D level. A 30-ng/mL cutoff point was selected a priori based on previous studies.^21^ Initial analyses testing for interaction between intervention group status and the dichotomized maternal 25(OH)D concentration variable did not show interactions at the *P* < .05 level (eAppendix in Supplement 2). However, exploratory post hoc linear and logistic regression analyses were conducted with adjustments as presented previously to examine differences in psychiatric problems among 4 subgroups created based on intervention group status and maternal 25(OH)D concentration. For reference groups, we first used the group with 400-IU supplementation and maternal 25(OH)D levels less than 30 ng/mL and then the group with 1200-IU supplementation and maternal 25(OH)D levels of 30 ng/mL or greater.

Analyses were conducted with sexes combined. Additional information on study characteristics stratified by sex and sex × intervention group interaction analysis is given in the eAppendix and eTables 3 and 4 in Supplement 2. Statistical analyses were performed using SPSS statistical software version 28 (IBM) and Stata statistical software version 17 (StataCorp). Data were analyzed from June 2022 to March 2023.

## Results

### Characteristics

The study population comprised 346 children (164 females [47.4%]; mean [SD] age at follow-up, 7.1 [0.4] years), including 169 children (80 females [47.3%]) in the 400-IU and 177 children (84 females [47.5%]) in the 1200-IU supplementation group. Table 1 presents baseline characteristics. Concentrations of 25(OH)D were significantly higher in the 1200-IU group compared with the 400-IU group at 1 year (MD, 13.2 ng/mL; 95% CI, 11.0 to 15.4 ng/mL; *P* < .001) and 2 years (MD, 12.7 ng/mL; 95% CI, 10.7 to 14.6 ng/mL; *P* < .001) (Table 2). Differences between groups were of the same magnitude as those seen for the original study population at 1 year (805 individuals) and 2 year (814 individuals) follow-up.^18^ In attrition analysis, there were no differences in externalizing or internalizing behavior at 2 years between participants in this study and nonparticipants with 2-year follow-up data (eAppendix in Supplement 2).

**Table 1.** Baseline Characteristics by Intervention Group

Characteristic	Families (N = 346)			
	Total with data, No.	400-IU vitamin D (n = 169) No. (%)	Total with data, No.	1200-IU vitamin D (n = 177) No. (%)
**Child**				
Sex				
Female	169	80 (47.3)	177	84 (47.5)
Male		89 (52.7)		93 (52.5)
Gestational length, mean (SD), d	169	280.4 (7.8)	177	281.2 (7.3)
Season of birth				
Winter	169	28 (16.6)	177	29 (16.4)
Spring		67 (39.6)		62 (35.0)
Summer		39 (23.1)		49 (27.7)
Autumn		35 (20.7)		37 (20.9)
**Parents**				
Mother				
Age at delivery, mean (SD), y	168	31.4 (4.0)	177	31.4 (4.3)
Smoking at childbirth	168	19 (11.3)	173	20 (11.6)
Pregnancy 25(OH)D concentration				
Mean (SD), ng/mL	143	33.7 (9.1)	148	33.4 (7.9)
<30 ng/mL	143	49 (29.0)	148	47 (26.6)
High educational level^a^	145	123 (84.8)	148	122 (82.4)
Depressive symptoms at childbirth, median (IQR), score^b^	155	11.0 (8.0-16.0)	163	10.0 (8.0-15.0)
CES-D score ≥16^c^	155	39 (25.2)	163	33 (19.6)
Father with high educational level	143	99 (69.2)	144	99 (68.8)

Abbreviations: 25(OH)D, serum 25-hydroxyvitamin D; CES-D, Center for Epidemiological Studies Depression Scale.

SI conversion factor: To convert 25(OH)D to nanomoles per liter, multiply by 2.496.

^a^ Parental education was categorized from 1 (comprehensive school) to 6 (university degree) and dichotomized into 2 levels: low (less than a bachelor’s degree) and high (bachelor’s degree or higher).

^b^ Depressive symptoms assessed using the CES-D.

^c^ Scores of 16 or greater indicate a risk of clinical depression.

**Table 2.** Follow-up Characteristics by Intervention Group

Characteristic	Families (N = 346)					
	Total with data, No.	400-IU vitamin D (n = 169) No. (%)	Total with data, No.	1200-IU vitamin D (n = 177) No. (%)	MD (95% CI)	*P* value^a^
At 12-mo follow-up						
Length of breastfeeding, mean (SD), mo	167	11.3 (5.7)	177	11.0 (5.5)	−0.3 (−1.5 to 0.9)	.59
25(OH)D concentration						
Mean (SD), ng/mL	160	34.4 (8.0)	164	47.6 (11.6)	13.2 (11.0 to 15.4)	<.001
<30 ng/mL	160	55 (34.4)	164	4 (2.4)	NA	<.001
At 24-mo follow-up						
25(OH)D concentration						
Mean (SD), ng/mL	168	35.5 (8.0)	175	48.1 (10.3)	12.7 (10.7 to 14.6)	<.001
<30 ng/mL	168	47 (30.0)	175	6 (3.4)	NA	<.001
At 6-8-y follow-up						
Age, mean (SD), y	169	7.2 (0.5)	177	7.1 (0.4)	−0.03 (−0.1 to 0.1)	.56
CBCL raw score, median (IQR)						
Internalizing problems	169	5.0 (2.0-8.0)	177	4.0 (2.0-7.0)	NA	.14
Externalizing problems		5.0 (2.0-10.0)		5.0 (3.0-9.0)	NA	.95
Total problems		20.0 (11.0-30.0)		20.0 (10.0-29.0)	NA	.71
CBCL *T* score, mean (SD)^b^						
Internalizing problems	169	51.0 (9.2)	177	49.5 (0.7)	−1.5 (−3.4 to 0.4)	.13
Externalizing problems		50.9 (8.8)		51.1 (9.0)	0.2 (−1.7 to 2.1)	.82
Total problems		49.8 (8.5)		49.4 (9.0)	−0.4 (−2.2 to 1.5)	.69
Clinically significant problems						
Internalizing problems	169	20 (11.8)	177	10 (5.6)	NA	.04
Externalizing problems		16 (9.5)		16 (9.0)	NA	.83
Total problems		9 (5.3)		8 (4.5)	NA	.90
Parent single at follow-up	146	16 (11.0)	153	7 (4.6)	NA	.04

Abbreviations: 25(OH)D, serum 25-hydroxyvitamin D; CBCL, Childhood Behavior Checklist; MD, mean difference; NA, not applicable.

SI conversion factor: To convert 25(OH)D to nanomoles per liter, multiply by 2.496.

^a^ *P* values calculated using χ^2^ test for proportions, Mann-Whitney *U* test for medians, *t* test for means (2-sided *P* values and equal variance not assumed), and linear regression (with maternal 25[OH]D level <30 ng/mL and 400-IU group as the reference).

^b^ CBCL raw scores converted to *T* scores and dichotomized. A *T* score of 64 or greater is considered to reflect clinically significant problems.

### Vitamin D_3_ Supplementation During Early Childhood and Psychiatric Problems at Ages 6 to 8 Years

There were 20 children (11.8%) in the 400-IU group and 10 children (5.6%) in the 1200-IU group with clinically significant internalizing problems (*P* = .04). The corresponding numbers for externalizing and total problems were 16 children (9.5%) vs 16 children (9.0%) (*P* = .83) and 9 children (5.3%) vs 8 children (4.5%) (*P* = .90), respectively (Table 2). The OR for clinically significant internalizing problems was 0.40 (95% CI, 0.17-0.94; *P* = .04) after adjustment for sex, birth season, maternal depressive symptoms at birth, and parental single status at follow-up. Results remained significant after correction for attrition bias. We conducted sensitivity analyses by repeating model 1 analyses and restricting the sample size to 318 children with available CES-D scores. Additionally, model 2 analyses were repeated using means substitution for 28 children missing CES-D scores to retain the entire sample in the analyses. Sensitivity analysis results were in line with primary results (eAppendix in Supplement 2). No effect of supplementation was found for externalizing (OR, 0.89; 95% CI, 0.42-1.91; *P* = .77) or total (OR, 0.81; 95% CI, 0.29-2.23; *P* = .68) problems (Table 3).

**Table 3.** Association Between Vitamin D Supplementation Group and Psychiatric Symptoms^a^

Measure	Comparison (95% CI)^b^	*P* value
**CBCL score, MD**		
Internalizing problems		
Model 1^c^	−0.17 (−0.38 to 0.04)	.12
Model 2^d^	−0.20 (−0.41 to 0.01)	.07
Externalizing problems		
Model 1^c^	0.03 (−0.18 to 0.24)	.79
Model 2^d^	0.01 (−0.21 to 0.22)	.94
Total problems		
Model 1^c^	−0.04 (−0.25 to 0.17)	.71
Model 2^d^	−0.08 (−0.29 to 0.14)	.48
**Clinically significant problems, OR**		
Internalizing problems		
Model 1^c^	0.45 (0.20 to 0.98)	.05
Model 2^d^	0.40 (0.17 to .0.94)	.04
Externalizing problems		
Model 1^c^	1.08 (0.53 to 2.20)	.83
Model 2^d^	0.89 (0.42 to 1.91)	.77
Total problems		
Model 1^c^	1.07 (0.42 to 2.69)	.90
Model 2^d^	0.81 (0.29 to 2.23	.68

Abbreviations: CBCL, Childhood Behavior Checklist; MD, mean difference; OR, odds ratio.

^a^ Psychiatric symptoms were assessed at ages 6 to 8 years using the Childhood Behavior Checklist.

^b^ In linear models, raw scores were square root transformed owing to skewness and converted to *Z* scores (0 = mean; 1 = 1 SD). MDs were calculated comparing children in the 1200-IU supplementation group with those in the 400 IU supplementation group. Raw scores were converted to *T* scores and dichotomized at 64 or greater to reflect clinically significant problems. ORs and 95% CIs from logistic regression analyses show odds of belonging to a group with clinically significant problems for the 1200-IU supplementation group compared with the 400-IU supplementation group.

^c^ Unadjusted model.

^d^ Model adjusted for sex, season of birth, maternal depressive symptoms at birth (using Center for Epidemiological Studies Depression Scale, and parental single status at follow-up. In adjusted models, there were 155 children for the 400-IU group and 163 children for the 1200-IU group owing to missing Center for Epidemiological Studies Depression Scale data.

Table 3 presents MDs for *Z* scores of square root–transformed internalizing (−0.20; 95% CI, −0.41 to 0.01; *P* = .07), externalizing (0.01; 95% CI, −0.21 to 0.22; *P* = .94), and total (−0.08; 95% CI, −0.29 to 0.14; *P* = .48) problem scores. Score distributions are presented in the eFigure in Supplement 2.

### Levels of 25(OH)D at 1 and 2 Years and Psychiatric Problems at Ages 6 to 8 Years

Higher 25(OH)D levels at ages 1 and 2 years resulted in lower risk for clinically significant internalizing problems (1 year: OR, 0.93; 95% CI, 0.90 to 0.97; *P* = .001; 2 years: OR, 0.95; 95% CI, 0.91 to 0.98; *P* = .01) and lower internalizing problem scores (1 year: MD, −0.010; 95% CI, −0.019 to −0.001; *P* = .04; 2 years: MD, −0.012; 95% CI, −0.021 to −0.002; *P* = .02) in the unadjusted model (eTable 5 in Supplement 2). After adjustment, the effect of 1-year 25(OH)D levels was attenuated for internalizing problem scores but remained for clinically significant internalizing problems (OR 0.94; 95% CI, 0.89 to 0.98; *P* = .01); the effect remained at 2 years for both outcomes after adjustment (internalizing problem score: MD, −0.011; 95% CI, −0.021 to −0.002; *P* = .02; internalizing problems: OR, −0.95; 95% CI, 0.91 to 0.99; *P* = .01). There was no effect of child 25(OH)D levels on externalizing or total problems.

### Maternal Vitamin D_3_, Early Childhood Supplementation, and Psychiatric Problems at Ages 6 to 8 Years

Among 291 families with data on maternal 25(OH)D levels, there were 96 children whose mothers had levels less than 30 ng/mL (33.0%) compared with 208 of 517 nonparticipating families with these data (40.2%). We found no effect of maternal 25(OH)D levels on child psychiatric problems (eTable 2 in Supplement 2). The proportion of children with clinically significant problems for each of 4 maternal 25(OH)D and infant vitamin D_3_ supplementation dose subgroups is given in eTable 6 in Supplement 2. The risk of clinically significant internalizing problems was significantly lower among 91 children in the 1200-IU group with maternal 25(OH)D levels of 30 ng/mL or greater compared with 44 children in the 400-IU group with maternal 25(OH)D levels less than 30 ng/mL (adjusted OR, 0.21; 95% CI, 0.06-0.78; *P* = .02) (Table 4).

**Table 4.** Association of Supplementation and Maternal Vitamin D Level Subgroup With Psychiatric Symptoms^a^

Measure	Group 2^b^^,^^c^		Group 3^b^^,^^c^		Group 4^b^^,^^c^	
	Comparison (95% CI)^d^	*P* value	Comparison (95% CI)^d^	*P* value	Comparison (95% CI)^d^	*P* value
**CBCL score, MD**						
Internalizing problems						
Model 1^e^	−0.39 (−0.80 to 0.01)	.05	−0.32 (−0.67 to 0.02)	.07	−0.41 (−0.75 to −0.07)	.02
Model 2^f^	−0.49 (−0.89 to −0.09)	.02	−0.30 (−0.66 to 0.05)	.10	−0.37 (−0.72 to −0.03)	.04
Externalizing problems						
Model 1^e^	−0.12 (−0.53 to 0.29)	.56	−0.21 (−0.56 to 0.14)	.24	−0.07 (−0.41 to 0.28)	.71
Model 2^f^	−0.12 (−0.54 to 0.29)	.56	−0.14 (−0.51 to 0.22)	.44	−0.02 (−0.38 to 0.34)	.91
Total problems						
Model 1^e^	−0.32 (−0.72 to 0.08)	.12	−0.41 (−0.76 to −0.06)	.02	−0.31 (−0.65 to 0.03)	.08
Model 2^f^	−0.34 (−0.75 to 0.06)	.10	−0.30 (−0.66 to 0.06)	.10	−0.23 (−0.58 to 0.12)	.19
**Clinically significant problems, OR**						
Internalizing problems						
Model 1^e^	0.30 (0.08 to 1.20)	.09	0.47 (0.17 to 1.28)	.22	0.23 (0.07 to 0.73)	.01
Model 2^f^	0.31 (0.07 to 1.31)	.11	0.50 (0.16 to 1.55)	.23	0.21 (0.06 to 0.78)	.02
Externalizing problems						
Model 1^e^	0.49 (0.15 to 1.60)	.33	0.49 (0.15 to 1.60)	.24	0.97 (0.34 to 2.75)	.95
Model 2^f^	0.28 (0.05 to 1.59)	.15	0.60 (0.17 to 2.19)	.44	1.02 (0.32 to 3.21)	.98
Total problems						
Model 1^e^	0.39 (0.07 to 2.12)	.28	0.39 (0.10 to 1.53)	.18	0.56 (0.16 to 1.92)	.35
Model 2^f^	0.19 (0.02 to 1.82)	.15	0.48 (0.11 to 2.12)	.34	0.53 (0.13 to 2.12)	.37

Abbreviations: ; CBCL, Childhood Behavior Checklist; MD, mean difference; OR, odds ratio.

^a^ Psychiatric symptoms were assessed at ages 6 to 8 years using the CBCL.

^b^ Groups 1 and 2 had maternal serum 25-hydroxyvitamin D (25[OH]D) levels less than 30 ng/mL (to convert to nanomoles per liter, multiply by 2.496), with child supplementation levels of 400-IU and 1200-IU vitamin D_3_, respectively. Groups 3 and 4 had maternal 25(OH)D levels of 30 ng/mL or greater, with child supplementation levels of 400-IU and 1200-IU vitamin D_3_, respectively.

^c^ Population numbers differ for model 1 vs model 2 analyses owing to missing data for the Center for Epidemiological Studies Depression Scale covariate (group 1: 49 vs 48 children; group 2: 47 vs 44 children; group 3: 94 vs 83 children; group 4: 101 vs 91 children).

^d^ In linear models, raw scores were square root transformed owing to skewness and converted to Z scores (0 = mean; 1 = 1 SD). MDs were calculated compared with children in group 1. In logistic regression models, raw scores were converted to *T* scores and dichotomized at 64 or greater to reflect clinically significant problems. ORs and 95% CIs from logistic regression analyses show odds of belonging to a group with clinically significant problems for each subgroup compared with the same reference group as described previously (group 1).

^e^ Unadjusted model.

^f^ Model adjusted for sex, season of birth, maternal depressive symptoms at birth, and parental single status at follow-up.

In a post hoc subgroup analysis, 48 children in the 400-IU group with maternal 25(OH)D concentrations less than 30 ng/mL had higher internalizing problems scores compared with children in the 1200-IU group, including 44 children whose mothers had 25(OH)D concentrations below (adjusted MD, 0.49; 95% CI, 0.09-0.89; *P* = .02) and 91 children whose mothers had concentrations above the 30-ng/mL cutoff (adjusted MD, 0.37; 95% CI, 0.03-0.72; *P* = .04) (Table 4). No significant differences were found between 400-IU and 1200-IU groups in children whose mothers had 25(OH)D concentrations of 30 ng/mL or greater (eTable 7 in Supplement 2).

## Discussion

In this secondary analysis of the VIDI RCT, we explored the potential impact of higher-than-standard vitamin D_3_ supplementation between ages 2 weeks and 2 years on psychiatric symptoms at mean age 7.1 years. We found a 5.6% prevalence of clinically significant internalizing problems in children who received 1200-IU oral vitamin D_3_ supplementation compared with 11.8% among those who received the standard recommended dose of 400 IU daily. No differences were found for total or externalizing problems. Although statistically significant differences at the *P* < .05 level were observed only for the dichotomous internalizing problems outcome, findings from the linear regression analysis followed the same direction, supporting the logistic regression findings.

Furthermore, internalizing problem scores were significantly higher for children from the 400-IU group with maternal 25(OH)D levels less than 30 ng/mL, compared with children from the 1200-IU group regardless of maternal 25(OH)D status. This should be interpreted cautiously, however, and be considered only as hypothesis generating given the absence of interactions between maternal 25(OH)D level and supplementation status.

Although previous studies^6,7,8,9,13,33^ have suggested that higher 25(OH)D levels during fetal life and early childhood may lower the risk of childhood psychopathology, to our knowledge, this is the first RCT to assess the potential impact of high-dose vitamin D_3_ supplementation in healthy infants and up to age 2 years on psychiatric symptoms during late preschool and early school age. We previously studied this population up to age 2 years and found no evidence of systematic benefits in child neurodevelopment of higher-than-standard supplementation.^10^ Conversely, a potential increase in externalizing problems among children in the 1200-IU supplementation group could not fully be excluded. In this study, we found no differences in externalizing problems between groups.

The potential associations of maternal and childhood 25(OH)D levels with later neurodevelopmental and mental health outcomes were previously comprehensively summarized.^6,7,34^ Only a few studies, however, explored the potential association between childhood vitamin D levels and features of internalizing behaviors, such as depression. In a prospective birth cohort study^8^ among 2759 children, higher 25(OH)D concentrations at mean age 9.8 years were associated with lower levels of depressive symptoms at age 13.8 years but not age 10.6 years, suggesting a sustained beneficial outcome increasing over time. A prospective cohort study^9^ among 273 children found that those with 25(OH)D levels less than 30 ng/mL (10.3% of participants) at age 5 to 12 years had higher internalizing and externalizing scores after a median follow-up of 6 years. Conversely, a 2022 cross-sectional study^35^ among 704 children and adolescents aged 11 to 16 years found no association between 25(OH)D concentration and depressive symptoms.

Studies focusing on maternal pregnancy 25(OH)D levels have reported inconsistent findings. A study^33^ among 487 mother-child pairs found an inverse association between first-trimester 25(OH)D levels and externalizing but not internalizing symptoms. In a pregnancy cohort study among 743 mother-child pairs, no associations were found between maternal 25(OH)D concentrations at the 18th pregnancy week and total, internalizing, or externalizing symptoms measured in the child at ages 2, 5, 8, 10, 14, or 17 years.^36^ In a study^13^ using data from VIDI and the Dutch Generation R cohort study (777 and 1505 mother-child pairs, respectively), lower early to midpregnancy 25(OH)D concentration was associated with higher infant negative affectivity. Negative affectivity is a temperament trait associated with increased risk for internalizing problems in childhood and adolescence.^37^ Although inconclusive, our subgroup analysis findings may suggest that exposure to maternal 25OHD levels lower than 30 ng/mL during pregnancy combined with standard dose supplementation could increase the risk for later internalizing problems compared with receiving higher-than-standard supplementation during early childhood and may potentially partly explain earlier inconsistencies in the literature.

### Strengths and Limitations

This study has several strengths, among them the double-blind RCT setting, standardized data collection, and well-characterized study population. Outcomes were assessed using CBCL, a widely used, validated questionnaire^38,39^ that allows for assessment of various aspects of childhood behavioral symptoms and potential emerging or manifest signs of psychopathology. CBCL was previously used in studies from Finland.^40,41^

This study has several limitations as well. Of the original study population (987 families), 546 families (55.3%) remained in the 6 to 8–year follow-up study, and of these, 346 families (63.4%) completed the CBCL questionnaire. Baseline characteristics were somewhat more beneficial among the study population compared with those lost to follow-up, potentially limiting generalizability to a more diverse population. Furthermore, the proportion of children with maternal 25(OH)D levels less than 30 ng/mL was lower among study participants than in nonparticipants (33.0% vs 40.2%), influencing the number of children available for subgroup analyses. However, attrition rates were similar between supplementation groups, baseline characteristics for nonparticipants did not differ between groups, and inverse probability weighting estimation revealed no indication of attrition bias. Externalizing and internalizing behaviors at age 2 years did not differ between participants and nonparticipants, suggesting that study participants were representative of the 2-year assessment participants in this regard.^10^

Questionnaires were collected from September 2020 to May 2021, concurrently with the global SARS-CoV-2 pandemic. The pandemic may have directly and indirectly negatively influenced mental health in children.^42,43^ We have no reason, however, to suspect that the burden of the pandemic would have differed between supplementation groups.

Since 2003, milk products and fat spreads in Finland have been fortified with vitamin D, resulting in improved population vitamin D levels.^44,45^ This may limit comparisons with studies performed in Finland before 2003 and with countries lacking systematic vitamin D fortification. Furthermore, whether our findings generalize to children living at other geographical latitudes needs to be investigated.

## Conclusions

This secondary analysis of an RCT found that a higher-than-standard vitamin D_3_ supplementation (1200 IU daily vs 400 IU) between ages 2 weeks and 2 years reduced the risk of internalizing problems later in childhood at ages 6 to 8 years. Results from the exploratory, post hoc subgroup analysis were inconclusive and need to be verified in future studies; further studies may suggest that early life higher-dose vitamin D_3_ supplementation is associated with benefits for children exposed to lower pregnancy 25(OH)D levels. Furthermore, study findings need to be interpreted in context with outcomes related to children’s somatic health (eg, growth and allergies), for which lower doses were found to be more beneficial during infancy^20,46^; these findings also need to be repeated and assessed for general safety.

## Notes

Supplement 1.
Trial Protocol and Statistical Analysis Plan

Supplement 2. **eAppendix.** Supplementary Methods
eReferences.
**eTable 1.** Attrition Table for Participants vs Nonparticipants **eTable 2.** Associations Between Covariates and Child Behavior Checklist Scores **eTable 3.** Study Participant Characteristics by Sex **eTable 4.** Child Psychiatric Symptoms and Problems by Sex **eTable 5.** Associations Between Vitamin D_3_ Concentrations at Ages 1 and 2 y and Psychiatric Symptoms at Ages 6 to 8 y **eTable 6.** Prevalence of Child Behavior Checklist *T* Scores within the Clinical Range for Maternal Vitamin D_3_ and Supplementation Status Subgroups **eTable 7.** Association of Subgroups by Vitamin D_3_ Supplementation and Maternal Vitamin D_3_ Level With Psychiatric Symptoms at Ages 6 to 8 y **eFigure.** Distribution of Raw Internalizing, Externalizing, and Total Problems Sum Scores by Supplementation Group

Supplement 3.
Data Sharing Statement
