# Supplementary material for: Effect of Vitamin D3 Supplementation in the First 2 Years of Life on Psychiatric Symptoms at Ages 6 to 8 Years: A Randomized Clinical Trial
Source: JAMA Netw Open. 2023 May 19;6(5):e2314319. doi: 10.1001/jamanetworkopen.2023.14319 (PMC10199342; doi:10.1001/jamanetworkopen.2023.14319)
Supplement: Supplement 1. — Trial Protocol and Statistical Analysis Plan [file jamanetwopen-e2314319-s001.pdf]

Supplementary online material

Supplement 1

Sandboge, S, Räikkönen K, Lahti-Pulkkinen M et. al. Effect of Vitamin D supplementation in the first 2 years on psychiatric symptoms at age 6-8 years: A randomized clinical

Pages 2-7 Original Trial Protocol and Statistical Analysis Plan

Pages 8-10 Amendments to the study protocol and statistical analysis plan included in *Effect of High-Dose vs Standard-Dose Vitamin D Supplementation on Neurodevelopment of Healthy Term Infants: A Randomized Clinical Trial* by Tuovinen et al

(*JAMA Netw Open.* 2021;4(9):e2124493. doi:10.1001/jamanetworkopen.2021.24493)

Pages 11-12 Amendments from the current study

PROTOCOL

Vitamin D Intervention in Infants (VIDI) is a large randomised trial that aims to evaluate effects of two vitamin D supplemental doses in early childhood on bone strength, infections, immunity, allergy, atopy and asthma, neurologic and cognitive development, and genetic regulation of mineral homeostasis.

This protocol comprises the applied methods to evaluate the effects of vitamin D supplementation on the primary outcomes of the study: bone strength and incidence of infections during the first two years of life. Methods for additional outcomes have been described in Helve et al, 2017.[1]

Background

Both cutaneously synthesised and vitamin D obtained from diet and supplements contribute to circulating 25-hydroxyvitamin D concentrations. The optimal 25-hydroxyvitamin D concentration is still under discussion. In 2011, Institute of Medicine guidelines stated that 25-hydroxyvitamin D concentrations above 50 nmol/L are required for normal body functions, including linear growth and bone mass accrual.[2] According to the Endocrine Society, concentrations above 75 nmol/L may be necessary to achieve optimal long-term health benefits.[3]

In Finland, Vitamin D supplementation has been recommended to all infants since the 1940's, but in line with the declining prevalence of rickets in our country, the recommended doses have gradually decreased. The present Finnish Nutritional Council guidelines recommend 10 µg (400 IU) of vitamin D3 supplementation daily for all infants from the age of 2 weeks to 2 years. Despite these recommendations, 20 % of 14-month-old children are shown to be vitamin D deficient (< 50 nmol/L).[4]

Normal bone development and growth requires adequate intake of minerals, such as calcium and phosphate. Parathyroid hormone and biologically active form of vitamin D, 1,25-dihydroxyvitamin D, regulate calcium and phosphate concentrations. In addition, 1,25-dihydroxyvitamin D has direct effect on bone cells. Insufficient vitamin D supply results in inadequate bone mineralisation at growth plates and leads to rickets.[5] In infants, severe vitamin D deficiency (< 25 nmol/L) associates with poor linear growth and delayed motor development possibly due to muscle weakness.[6,7] Correction of vitamin D deficiency has been shown to improve growth velocity and prevent stunted growth.[8,9] Data on the relationship between vitamin D and bone mineral density (BMD) in children are contradictory and further studies are essential.[10,11]

In addition to its skeletal effects, vitamin D modulates both the innate and adaptive immune systems. Immune cells express vitamin D receptor (VDR) and have local enzymatic capacity to synthetise active 1,25-dihydroxyvitamin D.[12,13] Vitamin D induces production of antimicrobial peptides, such as cathelicidin, by monocytes and macrophages, and is involved in regulation of T-lymphocyte function.[14]

In epidemiological studies, vitamin D deficiency has been associated with increased risk of infections both in adults and children.[15,16] Results from randomised trials are inconclusive. For example, in Japan, a daily supplemental vitamin D3 dose of 30 µg (1200 IU) for 4 months resulted in a significant reduction of influenza A infections, and in Mongolia, daily 7.5 µg (300 IU) of vitamin D3 reduced the risk of acute respiratory infections in schoolchildren.[17,18] However, in Afghan infants, the incidence of pneumonia was not affected by 18 months of vitamin D supplementation.[19] Further studies are needed to determine whether vitamin D supplementation provides benefits against infections in healthy children.

Primary outcomes

Primary outcomes of the study are bone strength measured by peripheral quantitative computed tomography (pQCT) and incidence of parent-reported infections at age 2 years.

Participants and methods

A total of 1 000 families are recruited and informed consent is obtained 1-2 days after the delivery at the Kätilöopisto Maternity Hospital in Helsinki. We include white northern European women with a singleton pregnancy and without regular medication. Healthy infants born at term (37-42 weeks) and with weight appropriate for gestational age are included in the study. Exclusion criteria for the infants are: seizures, need for early antibiotic

treatment, need for nasal continuous positive airway pressure > 24 hours, extended phototherapy > 72 hours, need for nasogastric tube > 24 hours or intravenous glucose infusion. Data on family background (parents' socio-economic status, health, lifestyle factors) and maternal dietary status are documented with a questionnaire. At birth, a cord blood sample (20 ml) is taken and stored for later analyses.

Participating infants are randomised to receive either the currently recommended vitamin D3 supplementation of 10 µg (400 IU) daily or a higher dose of 30 µg (1200 IU) daily from age 2 weeks to 2 years. Boys and girls are randomised separately in blocks of 50. Randomisation is performed by the Helsinki University Hospital Pharmacy. The study is double-blinded. Vitamin D is administered orally as vitamin D3, with a dose of 5 drops a day for both concentrations.

Follow-up

Participants are assessed at a study outpatient clinic by a study nurse and/or pediatrician at 6 months, 1 year and 2 years of age. Growth parameters (length, weight, head circumference) are measured and compared with Finnish growth charts.[20] Blood samples are taken for biochemistry. Bone strength of participants is evaluated by pQCT at 1 and 2 years of age. Families are provided study diaries where they keep daily records on dosing of vitamin D3 supplement and on all infections of the participating child. Nutrient intake from food is evaluated from 3-day food record at 1 year and a Food Frequency Questionnaire (FFQ) at 2 years of age.

Specific methods

Biochemical markers

Serum 25-hydroxyvitamin D concentration is measured from serum samples with an automated IDS-iSYS analyser (IDS Ltd., Bolton, UK) which employs a chemiluminescence immunoassay (CLIA) with high sensitivity, a fast protocol, with a 10-µl specimen volume. The method is validated against LC-MS in-house as well as by the manufacturer. Analyses are performed at the Pediatric Research Centre laboratory in Biomedicum, University of Helsinki (Helsinki, Finland). Reproducibility is ensured by adhering to the Vitamin D External Quality Assessment Scheme (DEQAS, Charing Cross Hospital, London UK). The IDS-iSYS immunoassay will also be used to analyse serum intact parathyroid hormone concentration from serum samples.

Ionised calcium (adjusted to pH 7.40, normal range 1.16-1.39 and 1.17-1.35 mmol/L for age groups 1-12 months and 24 months respectively) is analysed from capillary blood samples at 6 months and from serum samples at 12 and 24 months at the Central Laboratory of Helsinki University Hospital (HUSLAB) using ABL 90 FLEX or ABL 835 FLEX blood gas analysers. HUSLAB is an accredited laboratory adhering to international (T055) SFS-EN ISO 15189 and SFS-EN ISO/IEC 17025 standards.

Bone strength

Bone strength is measured by pQCT from the distal left tibia using a XCT-2000 scanner (Stratec Medizintechnik GmbH, Pforzheim, Germany).

Infections

Data on infections are collected prospectively from daily diaries kept by parents of participating infants. Parents record the time and duration of the infection, symptoms, required medication, physician visit or hospitalisation in the daily diaries. Every 3-6 months, new diaries are provided to the families by mail or at follow-up visits, and completed diaries are returned to the study nurse.

Dietary vitamin D intake

At recruitment, maternal diet is evaluated retrospectively with a semi quantitative 22-item Food Frequency Questionnaire, for the time-period of one month before delivery. At age 1 years, data on nutrient intake from food is collected with a three-day food record and analysed using AivoDiet software (Aivo Oy Finland, Turku, Finland). At 2 years, the dietary habits of the child are collected with 47-item Food Frequency Questionnaire. If the child attends daycare, the personnel are asked to complete a separate two-day food record.

Ethical issues and research permits

A recent intervention study confirmed the safety of vitamin D supplementation in infants with a daily dose of 50 µg (2000 IU) daily.[21] Furthermore, we conducted a pilot study in 113 healthy newborns in order to evaluate short-term effects and safety of 3 different vitamin D3 doses (10 µg, 30 µg, 40 µg daily).[22] No adverse events occurred and all doses were deemed safe. Based on the results, we chose daily doses of 10 µg and 30 µg for the intervention study.

An external clinical research institute monitors the study and possible adverse effects. As a safety protocol, the infants are monitored for hypercalcemia at follow-up visits. If the calcium concentration exceeds the upper reference limit of ionised calcium by ≥10%, defined as ionised calcium concentration above 1.53 mmol/L at 6 and 12 months and 1.48 mmol/L at 2 years follow-up, the ionised calcium and 25-hydroxyvitamin D concentrations will be repeatedly measured, symptoms indicative of hypercalcemia will be evaluated, and, if necessary, dosing of vitamin D supplementation adjusted.

Blood samples are taken as follows: A) 20 ml from the umbilical vein after cord clamping at birth, B) 15 ml at age 1 year, and C) ≤ 20 ml at age 2 years. These volumes are clearly below the allowed maximal volumes for research sampling (approximate limits for research purposes are for ages 1 year: 24 ml and 2 years: 36 ml; i.e. 3% of circulating blood volume).

The radiation exposure from pQCT measurements is estimated to be 30 µSv, and exposure from whole-body DXA 50 µSv. This total dose of approximately 80 µSv equals radiation exposure during an overseas flight or 2 weeks' background radiation, and can thus be regarded as insignificant.[23] Similar methods have previously been used to study bone variables in newborns.[22]

The vitamin D3 supplements are provided by Orion Pharmaceuticals free of charge. The study is researcher initiated and independent.

Informed consent is obtained from the parents at recruitment. An ethical approval has been obtained from the Research Ethics Committee of the Hospital District of Helsinki and Uusimaa (ID 107/13/03/03/2012) including permission to keep a research register of collected data where the anonymity of all participants is secured with an identification number. Research permits from Children's Hospital are valid until 2018. The project protocol is registered into ClinicalTrial.com (NCT01723852).

References

1. Helve O, Viljakainen H, Holmlund-Suila E, et al. Towards evidence-based vitamin D supplementation in infants: vitamin D intervention in infants (VIDI) - study design and methods of a randomised controlled double-blinded intervention study. BMC Pediatr 2017;17:91,017-0845-5.

2. Ross AC. The 2011 report on dietary reference intakes for calcium and vitamin D. Public Health Nutr 2011;14:938-9.

3. Holick MF, Binkley NC, Bischoff-Ferrari HA, et al. Guidelines for preventing and treating vitamin D deficiency and insufficiency revisited. J Clin Endocrinol Metab 2012;97:1153-8.

4. Viljakainen HT. Maternal vitamin D status affects bone growth in early childhood-a prospective cohort study. Osteoporosis Int 2011;22:883-91.

5. Elder CJ, Bishop NJ. Rickets. Lancet 2014;383:1665-76.

6. Pettifor JM, Prentice A. The role of vitamin D in paediatric bone health. Best Pract Res Clin Endocrinol Metab 2011;25:573-84.

7. Soliman A, Salama H, Alomar S, Shatla E, Ellithy K, Bedair E. Clinical, biochemical, and radiological manifestations of vitamin D deficiency in newborns presented with hypocalcemia. Indian J Endocrinol Metab 2013;17:697-703.

8. Soliman AT, El-Dabbagh M, Adel A, Al Ali M, Aziz Bedair EM, Elalaily RK. Clinical responses to a mega-dose of vitamin D3 in infants and toddlers with vitamin D deficiency rickets. J Trop Pediatr 2010;56:19-26.

9. Kumar GT, Sachdev HS, Chellani H, et al. Effect of weekly vitamin D supplements on mortality, morbidity, and growth of low birthweight term infants in India up to age 6 months: randomised controlled trial. BMJ 2011;342:d2975.

10. Viljakainen HT, Saarnio E, Hytinantti T, et al. Maternal vitamin D status determines bone variables in the newborn. J Clin Endocrinol Metab 2010;95:1749-57.

11. Mayranpaa MK, Tamminen IS, Kroger H, Makitie O. Bone biopsy findings and correlation with clinical, radiological, and biochemical parameters in children with fractures. J Bone Miner Res 2011;26:1748-58.

12. Holick MF. Vitamin D deficiency. N Engl J Med 2007;357:266-81.

13. Di Rosa M, Malaguarnera M, Nicoletti F, Malaguarnera L. Vitamin D3: a helpful immuno-modulator. Immunology 2011;134:123-39.

14. Hewison M. An update on vitamin D and human immunity. Clin Endocrinol (Oxf) 2012;76:315-25.

15. Ginde AA, Camargo CA,Jr, Shapiro NI. Vitamin D insufficiency and sepsis severity in emergency department patients with suspected infection. Acad Emerg Med 2011;18:551-4.

16. McNally JD, Leis K, Matheson LA, Karuananyake C, Sankaran K, Rosenberg AM. Vitamin D deficiency in young children with severe acute lower respiratory infection. Pediatr Pulmonol 2009;44:981-8.

17. Urashima M, Segawa T, Okazaki M, Kurihara M, Wada Y, Ida H. Randomized trial of vitamin D supplementation to prevent seasonal influenza A in schoolchildren. Am J Clin Nutr 2010;91:1255-60.

18. Camargo CA,Jr, Ganmaa D, Frazier AL, et al. Randomized trial of vitamin D supplementation and risk of acute respiratory infection in Mongolia. Pediatrics 2012;130:e561-7.

19. Manaseki-Holland S, Maroof Z, Bruce J, et al. Effect on the incidence of pneumonia of vitamin D supplementation by quarterly bolus dose to infants in Kabul: a randomised controlled superiority trial. The Lancet 2012;379:1419-27.

20. Saari A, Sankilampi U, Hannila ML, Kiviniemi V, Kesseli K, Dunkel L. New Finnish growth references for children and adolescents aged 0 to 20 years: Length/height-for-age, weight-for-length/height, and body mass index-for-age. Ann Med 2011;43:235-48.

21. Wicklow BA, Taback SP. Feasibility of a type 1 diabetes primary prevention trial using 2000 IU vitamin D3 in infants from the general population with increased HLA-associated risk. Ann N Y Acad Sci 2006;1079:310-2.

22. Holmlund-Suila E, Viljakainen H, Hytinantti T, Lamberg-Allardt C, Andersson S, Makitie O. High-dose vitamin d intervention in infants--effects on vitamin d status, calcium homeostasis, and bone strength. J Clin Endocrinol Metab 2012;97:4139-47.

23. Blake GM, Naeem M, Boutros M. Comparison of effective dose to children and adults from dual X-ray absorptiometry examinations. Bone 2006;38:935-42.

STATISTICAL ANALYSIS PLAN

Sample size calculation and statistical analyses

We aim at detecting a 0.2 SD unit difference between continuous bone strength parameters; bone mineral content and cross-sectional area. In order to reach a statistical power of 90% with significance level of 0.05, a total of 210 and 297 measurements, respectively, are necessary.[1] In addition, challenges in measuring infants with pQCT may occur. According to previous studies, small children suffer from an average of 6 infections annually.[2,3] In order to detect a decrease from 12 to 9 infections during the 24-month study period, a sample size of 220 in each group is required. We estimate a possible drop-out rate of 20%. Taking into consideration all the aforementioned aspects, the trial is designed for 1000 subjects: 500 subjects in each intervention group.

Statistical methods

Comparisons between two intervention groups are analysed with independent samples t-test, Mann Whitney U-test or Pearson Chi-square as applicable. If possible, logarithmic transformation for non-normal variables is performed in order to achieve normal distribution. The impact of vitamin D supplementation, and relevant covariates, on serum 25-hydroxyvitamin D concentration is analysed with linear mixed model. For continuous variables (bone strength), differences between intervention groups are assessed with multivariate analysis of covariance (MANCOVA). For evaluating the effect of vitamin D supplementation on the frequency of infections, negative binomial model is applied. Infection incidence is estimated as proportion of follow-up time as person-months allowing utilisation of all available data. Main outcome measures are analysed according to intention-to-treat and protocol-based manner.

References

1. Ireland A, Rittweger J, Schönau E, Lamberg-Allardt C, Viljakainen H. Time since onset of walking predicts tibial bone strength in early childhood. Bone. 2014 Nov;68:76-84.

2. Wald ER, Guerra N, Byers C. Frequency and severity of infections in day care: three-year follow-up. J Pediatr 1991;118:509-14.

3. Denny FW, Collier AM, Henderson FW. Acute respiratory infections in day care. Rev Infect Dis 1986;8:527-32.

AMENDMENTS TO THE STUDY PROTOCOL AND STATISTICAL ANALYSIS PLAN INCLUDED IN *EFFECT OF HIGH-DOSE VS STANDARD-DOSE VITAMIN D SUPPLEMENTATION ON NEURODEVELOPMENT OF HEALTHY TERM INFANTS: A RANDOMIZED CLINICAL TRIAL* BY TUOVINEN ET AL

PROTOCOL

Background

Lower vitamin D concentrations are associated with neurodevelopmental disorders, including autism spectrum disorder (ASD) and attention-deficient hyperactivity disorder (ADHD) in children.[1,2] Associations between vitamin D concentrations and cognitive and motor functioning has not been systematically found.[3] However, previous evidence is based on observational studies and no causal relationship can be inferred. Randomized controlled trials (RCT),[4–7] as well as non-randomized trials,[8–12] on vitamin D supplementation in children have been small-scale studies, focused typically on symptom severity among children with ASD or ADHD, and reported mixed findings. Further studies are needed to test whether vitamin D supplementation from infancy on in healthy community-based children provides benefits on neurodevelopment.

Primary outcomes

Primary outcomes of the study are developmental milestones measured with the Ages and Stages Questionnaire (ASQ) at age 12 and 24 months and social-emotional problems and competencies measured with Infant-Toddler Social Emotional Assessment (ITSEA) at age 24 months.

Follow-up

Participants are assessed at a study outpatient clinic by a study nurse and/or pediatrician at 12 months and 24 months of age. Data on developmental milestones are collected with Ages and Stages Questionnaire (ASQ)[13,14] at 12- and 24-month and data on social-emotional problems and competencies with Infant-Toddler Social Emotional Assessment (ITSEA)[15] at 24-month follow-ups. Blood samples are taken for biochemistry. Families are provided study diaries where they keep daily records on dosing of vitamin D3 supplement.

Specific methods

Developmental milestones

Developmental milestones are assessed with parent-reported Ages and Stages Questionnaire (ASQ) 3rd edition. ASQ is a reliable and valid tool with high sensitivity and specificity for screening children requiring further developmental assessment.[13,14] It comprises 21 age-specific questionnaires, covering 1 through 66 months of age, each with six items in each of five developmental domains: communication, gross motor, fine motor, problem solving, and personal/social (solitary social play and play with toys and other children) skills.[13] Subscale scores range from 0 to 60. We chose the 12-month (11-13 months) and 24-month (23-25.5 months) questionnaires.

Social-emotional problems and competencies

Social-emotional problems and competencies are assessed with parent-reported Infant-Toddler Social Emotional Assessment (ITSEA). It is an adult-report questionnaire for 12-to 36-month olds.[15] It includes 169 items containing a statement about the child’s behavior during the last month. The scale has good psychometric properties.[15]

STATISTICAL ANALYSIS PLAN

Sample size calculation and statistical analyses

We aim at detecting a 0.2 SD unit difference between continuous variables (developmental milestones and social-behavioral problems and competencies) and Odds Ratios >2.0 in categorical variables (mild developmental delay, clinically possible significant problems).[16] In order to reach statistical power of 80% with significance level of 0.05, a total of 199 and 572 participants, respectively, are necessary. The trial was designed with a planned sample of 1000 study participants. We estimate a possible drop-out rate of 20% for each follow-up.

Statistical methods

Standard statistical methods such as linear, logistic and Tobit and moderation analytic strategies with R/SPSS/MPlus will be used to study the impact of vitamin D supplementation and/or 25(OH)D concentrations, and relevant covariates, on main outcomes.

If needed, logarithmic transformation for nonnormal variables is performed in order to achieve normal distribution. Main outcome measures are analysed according to intention-to-treat and protocol-based manner.

To account for multiple testing and control for the false detection rate (FDR), *p*-values from the analysis within primary/secondary analysis were used to derive an FDR-adjusted *p*-values with FDR-procedure setting the false discovery rate at 0.05.[17]

REFERENCES

1. Föcker M, Antel J, Ring S, et al. Vitamin D and mental health in children and adolescents. Eur Child Adolesc Psychiatry. 2017;26:1043-1066. doi:10.1007/s00787-017-0949-3

2. Khoshbakht Y, Bidaki R, Salehi-Abargouei A. Vitamin D status and attention deficit hyperactivity disorder: A systematic review and meta-analysis of observational studies. Adv Nutr. 2018;9:9-20. doi:10.1093/advances/nmx002

3. Mutua AM, Mogire RM, Elliott AM, et al. Effects of vitamin D deficiency on neurobehavioural outcomes in children: A systematic review. Wellcome Open Res. 2020;5:28. doi:10.12688/wellcomeopenres.15730.1

4. Mohammadpour N, Jazayeri S, Tehrani-Doost M, et al. Effect of vitamin D supplementation as adjunctive therapy to methylphenidate on ADHD symptoms: A randomized, double blind, placebo-controlled trial. Nutr Neurosci. 2018. doi:10.1080/1028415X.2016.1262097

5. Saad K, Abdel-Rahman AA, Elserogy YM, et al. Randomized controlled trial of vitamin D supplementation in children with autism spectrum disorder. J Child Psychol Psychiatry Allied Discip. 2018. doi:10.1111/jcpp.12652

6. Kerley CP, Power C, Gallagher L, Coghlan D. Lack of effect of Vitamin D 3 supplementation in autism: A 20-week, placebo-controlled RCT. Arch Dis Child. 2017. doi:10.1136/archdischild-2017-312783

7. Azzam HME, Sayyah H, Youssef S, et al. Autism and vitamin D: An intervention study. Middle East Curr Psychiatry. 2015. doi:10.1097/01.XME.0000457269.05570.78

8. Elshorbagy HH, Barseem NF, Abdelghani WE, et al. Impact of Vitamin D Supplementation on Attention-Deficit Hyperactivity Disorder in Children. Ann Pharmacother. 2018. doi:10.1177/1060028018759471

9. Saad K, Abdel-rahman AA, Elserogy YM, et al. Vitamin D status in autism spectrum disorders and the efficacy of vitamin D supplementation in autistic children. Nutr Neurosci. 2016. doi:10.1179/1476830515Y.0000000019

10. Feng J, Shan L, Du L, et al. Clinical improvement following vitamin D3 supplementation in Autism Spectrum Disorder. Nutr Neurosci. 2017. doi:10.1080/1028415X.2015.1123847

11. Ucuz II, Dursun OB, Esin IS, et al. The relationship between Vitamin D, autistic spectrum disorders, and cognitive development: Do glial cell line-derived neurotrophic factor and nerve growth factor play a role in this relationship? Int J Dev Disabil. 2015;6:222-230. doi:10.1179/2047387714Y.0000000054

12. Jia F, Wang B, Shan L, Xu Z, Staal WG, Du L. Core symptoms of autism improved after vitamin D supplementation. Pediatrics. 2015;135:e196-e198. doi:10.1542/peds.2014-2121

13. Squires J, Bricker D. Ages and Stages Questionnaire (ASQ): A Parent Completed Child System (3rd Ed.). Baltimore: MD: Brooks Publishing Company; 2009.

14. Squires J, Bricker D, Potter L. Revision of a parent-completed developmental screening tool: Ages and stages questionnaires. J Pediatr Psychol. 1997;22(3):313-328. doi:10.1093/jpepsy/22.3.313

15. Briggs-Gowan M, Carter AS. Preliminary acceptability and psychometrics of the Infant–Toddler Social and Emotional Assessment (ITSEA): A new adult-report questionnaire. Infant Ment Health J. 1998;19:422-445.

16. Chen H, Cohen P, Chen S. How big is a big odds ratio? Interpreting the magnitudes of odds ratios in epidemiological studies. Commun Stat Simul Comput. 2010;39:860-864. doi:10.1080/03610911003650383

17. Benjamini Y, Hochberg Y. Controlling the false discovery rate: a practical and powerful approach to multiple testing. J R Stat Soc. 1995;57:289-300.

AMENDMENTS TO THE STUDY PROTOCOL AND STATISTICAL ANALYSIS PLAN FROM THE CURRENT STUDY

PROTOCOL

Background

Previous studies have linked lower childhood 25(OH)D levels to increased depressive symptoms,[1] as well as to increased internalizing, and externalizing problems,[2] in later childhood. Psychiatric problems may not be manifest during early childhood but become evident with age as environmental demands increase.[3] Against this background, the aim of this follow-up study was to build upon our previous study and assess the impact of higher dose vitamin D_3 supplementation on psychiatric symptoms at ages 6-8 years, an age period characterized by increased demands for self-regulation in concurrence with a still developing prefrontal cortex.[4]

Lower maternal 25(OH)D levels during pregnancy have also been linked to unfavorable offspring mental health outcomes, e.g., increased negative affectivity in infancy,[5] and depression.[6] Our secondary aim was to assess whether a higher-than-normal vitamin D_3 supplementation during infancy might mitigate the potential negative impact of lower pregnancy 25(OH)D levels.

Primary outcomes

Primary outcomes of the study are childhood psychiatric symptoms at ages 6-8 years, assessed using the parent-reported Child Behavior Checklist (CBCL).[7]

Follow-up

Parents completed the 113 question CBCL questionnaire at child ages 6-8 years.

Specific methods

Psychiatric symptoms

Psychiatric symptoms were assessed using the parent reported CBCL questionnaire. Results from the questionnaire are used to calculate scores for internalizing, and externalizing symptoms as well as total problems. Using ASEBA software, scores are converted to sex- and age-specific T-scores and dichotomized at 64 or above – scores above the cutoff point are considered to reflect clinically significant problems.

STATISTICAL ANALYSIS PLAN

Statistical methods

Standard statistical methods such as linear and logistic regression with SPSS software will be used to study the impact of vitamin D supplementation and/or maternal 25(OH)D concentrations, and relevant covariates, on main outcomes.

If needed, logarithmic transformation will be used to normalize skewed variables. Outcome measures will be analyzed according to the intention-to-treat principle.

REFERENCES

1. Tolppanen AM, Sayers A, Fraser WD, Lewis G, Zammit S, Lawlor DA. The association of serum 25-hydroxyvitamin D3 and D2 with depressive symptoms in childhood--a prospective cohort study. *J Child Psychol Psychiatry*. 2012;53(7):757-766. doi:10.1111/j.1469-7610.2011.02518.x

2. Robinson SL, Marín C, Oliveros H, Mora-Plazas M, Lozoff B, Villamor E. Vitamin D Deficiency in Middle Childhood Is Related to Behavior Problems in Adolescence. *J Nutr*. 2020;150(1):140-148. doi:10.1093/jn/nxz185

3. Solmi M, Radua J, Olivola M, et al. Age at onset of mental disorders worldwide: large-scale meta-analysis of 192 epidemiological studies. *Mol Psychiatry*. 2022;27(1):281-295. doi:10.1038/s41380-021-01161-7

4. Eyles DW, Feron F, Cui X, et al. Developmental vitamin D deficiency causes abnormal brain development. *Psychoneuroendocrinology*. 2009;34 Suppl 1:S247-257. doi:10.1016/j.psyneuen.2009.04.015

5. Sammallahti S, Holmlund-Suila E, Zou R, et al. Prenatal maternal and cord blood vitamin D concentrations and negative affectivity in infancy. *Eur Child Adolesc Psychiatry*. Published online October 18, 2021. doi:10.1007/s00787-021-01894-4

6. Strøm M, Halldorsson TI, Hansen S, et al. Vitamin D measured in maternal serum and offspring neurodevelopmental outcomes: a prospective study with long-term follow-up. *Ann Nutr Metab*. 2014;64(3-4):254-261. doi:10.1159/000365030

7. Achenbach T. Manual for The Child Behavior Checklist/4-18 and 1991 Profile. *Univ Vt Dep Psychiatry*. Published online 1991. Accessed October 9, 2022. https://cir.nii.ac.jp/crid/1570009749384841472
