# Supplementary material for: Effect of Vitamin D3 Supplementation in the First 2 Years of Life on Psychiatric Symptoms at Ages 6 to 8 Years: A Randomized Clinical Trial
Source: JAMA Netw Open. 2023 May 19;6(5):e2314319. doi: 10.1001/jamanetworkopen.2023.14319 (PMC10199342; doi:10.1001/jamanetworkopen.2023.14319)
Supplement: Supplement 2. — eAppendix. Supplementary Methods eReferences. eTable 1. Attrition Table for Participants vs Nonparticipants eTable 2. Associations Between Covariates and Child Behavior Checklist Scores eTable 3. Study Participant Characteristics by Sex eTable 4. Child Psychiatric Symptoms and Problems by Sex eTable 5. Associations Between Vitamin D3 Concentrations at Ages 1 and 2 y and Psychiatric Symptoms at Ages 6 to 8 y eTable 6. Prevalence of Child Behavior Checklist T Scores within the Clinical Range for Maternal Vitamin D3 and Supplementation Status Subgroups eTable 7. Association of Subgroups by Vitamin D3 Supplementation and Maternal Vitamin D3 Level With Psychiatric Symptoms at Ages 6 to 8 y eFigure. Distribution of Raw Internalizing, Externalizing, and Total Problems Sum Scores by Supplementation Group [file jamanetwopen-e2314319-s002.pdf]

Supplemental Online Content

Sandboge S, Räikkönen K, Lahti-Pulkkinen M, et al. Effect of vitamin D_3 supplementation in the first 2 years of life on psychiatric symptoms at ages 6 to 8 years: a randomized clinical trial. *JAMA Netw Open*. 2023;6(5):e2314319. doi:10.1001/jamanetworkopen.2023.14319

eAppendix. Supplementary Methods
eReferences.
eTable 1. Attrition Table for Participants vs Nonparticipants
eTable 2. Associations Between Covariates and Child Behavior Checklist Scores
eTable 3. Study Participant Characteristics by Sex
eTable 4. Child Psychiatric Symptoms and Problems by Sex
eTable 5. Associations Between Vitamin D_3 Concentrations at Ages 1 and 2 y and Psychiatric Symptoms at Ages 6 to 8 y
eTable 6. Prevalence of Child Behavior Checklist *T* Scores Within the Clinical Range for Maternal Vitamin D_3 and Supplementation Status Subgroups
eTable 7. Association of Subgroups by Vitamin D_3 Supplementation and Maternal Vitamin D_3 Level With Psychiatric Symptoms at Ages 6 to 8 y
eFigure. Distribution of Raw Internalizing, Externalizing, and Total Problems Sum Scores by Supplementation Group

This supplemental material has been provided by the authors to give readers additional information about their work.

© 2023 Sandboge S et al. *JAMA Network Open*.

eAppendix. Supplementary Methods

Baseline family characteristics and covariate creation
The participating families filled out self-administered research questionnaires on health and lifestyle factors at recruitment. Electronic hospital records were used to obtain data on gestation, delivery, and infant demographics. The month of birth was categorized according to season: winter (December, January, February), spring (March, April, May), summer (June, July, August), and autumn (September, October, November). Parental education was categorized from 1 (comprehensive school) to 6 (university degree) and dichotomized into two levels, low (less than a bachelor's degree) and high (bachelor's degree or above) educational levels. Information on smoking status before pregnancy and after delivery was collected through questionnaires and used to create a dichotomous variable. Missing values for maternal education and smoking status were dummy coded into their own categories. The duration of breastfeeding was recorded in study diaries. Maternal depressive symptoms at birth were assessed using the Center for Epidemiological Studies Depression Scale (CES-D),[1] a 20-item measure of depression with each item scored from 0 (rarely, none of the time) to 3 (most or almost all the time) resulting in a 0-60 score range. Scores of 16 or above reflect moderate depressive symptomatology while scores of 24 or above reflect severe symptomatology. In the current study, scores were square root transformed due to skewness before inclusion in regression models. Information regarding parental single status were collected through questionnaires at the 6–8-year follow-up and used to create a dichotomous variable. Missing values were dummy coded into their own category.

Biochemical analyses
Maternal serum samples were collected as part of routine maternity clinic follow-up visits at 6-27 weeks of gestation (mean = 11.3, standard deviation (SD) = 1.9) and stored in the Finnish Maternity Cohort serum bank, organized by the National Institute for Health and Welfare. Childhood serum samples were collected at ages one and two years. The IDS-iSYS fully automated immunoassay system with chemiluminescence detection (Immunodiagnostic Systems Ltd., Bolton, UK) was used in the analysis of 25-hydroxyvitamin D. The method has been demonstrated to have a good linear agreement with liquid chromatography in tandem with mass spectroscopy (LC-MS, R^2=0.942, in-house comparison of 67 samples). Mean (95% Confidence Interval (CI) value for the ratio of IDS-iSYS 25(OH)D to LC-MS 25(OH)D is 0.73 (0.68; 0.78) while intra-assay variations were 7%. Analysis took place at the Pediatric Research Centre, University of Helsinki. Our laboratory participates in the inter-laboratory quality assessment scheme for vitamin D, DEQAS (Charing Cross Hospital, London, UK).

Attrition analysis
In an attrition analysis, we found that children who did not take part in the current study (n = 628), either due to non-participation in VIDI2 or non-completion of the CBCL questionnaire, had parents with lower attained education, and mothers with lower mean 25(OH)D concentrations during pregnancy (32.3 ng/mL vs 33.6 ng/mL), who smoked more often (20% vs 14%) and had shorter length of breastfeeding (10.3 vs 11.2 months), compared to those who took part in the follow-up (**eTable 1**). Differences between non-participants and participants did not vary between supplementation groups and the attrition rate was similar. 13 children were not included in the attrition analysis, 12 of whom did not fulfil inclusion criteria and one who was diagnosed with a rare genetic disorder after study recruitment

To account for potential attrition bias regarding internalizing and/or externalizing behavior between participants and non-participants, we compared the rank-normalized values, according to the Blom formula,[2] of subscales from Infant-Toddler Social and Emotional Assessment (ITSEA) questionnaire assessed in our previous study at 2 years of age.[3] There were no significant differences between children lost to follow-up and children participating in the current study (Mean difference (MD) 0.03 (95% CI -0.12 to 0.18, *P* = .69 for externalizing behavior) MD 0.02 (95% CI -0.14 to 0.17, *P* = .82) for internalizing behavior).

Sensitivity analysis
To assess the potential impact of missing data, we reran the Model 1 analysis excluding individuals lacking data on maternal depressive symptoms (n = 28). For internalizing problems, the regression coefficient in the linear model using this approach was -0,21 (95% CI; -0.43 to 0.01, p = .07) and the OR for clinically significant problems was 0.42 (95% CI 0.18 to 0.96, p = .04), i.e., of the same significance level, magnitude, and direction as in the full sample. A similar lack of difference between the larger and smaller sample size was seen for externalizing, and total problems. Likewise, when using means substitution for those missing CES-D data to retain the entire sample in the analysis, findings for Model 2 were similar as those currently reported. For example, the regression coefficient in the linear model using this approach was -0,16 (95% CI; -0.37 to 0.05, p =

© 2023 Sandboge S et al. *JAMA Network Open*.

.14) and the OR for clinically significant problems was 0.44 (95% CI 0.19 to 0.98, p = .04). In other words, the smaller sample size in Model 2 did most likely not influence/bias the results in a meaningful manner.

Inverse probability weighting analyses testing the impact of attrition

Inverse probability weighting (IPW) was used to assess whether findings might have been impacted by attrition bias. The probability of taking part in the 6–8-year follow-up study was assessed as a function of baseline covariates associated with non-participation using logistic regression. We then estimated the association between vitamin D supplementation and clinically significant internalizing problems at age 6-8 years weighting the participants by the inverse of their probability of follow-up participation. Running IPW estimation, children in the 1200 IU supplementation group had a 0.07 (95% CI 0.00 to 0.14; *P*=.04) lower probability of having clinically significant internalizing problems compared to children in the 400 IU group, corresponding to an OR of 0.40 (95% CI 0.17 to 0.95; *P*=.04), i.e., of the same magnitude as in the main analysis, ruling out attrition bias.

Interaction

To explore the potential interplay between supplementation status and maternal pregnancy vitamin D status, the supplementation groups were further stratified by maternal 25(OH)D status. A 30 ng/mL cutoff point was chosen a priori, based upon previous literature.[4] An interaction term was calculated by multiplying supplemental group status with maternal 25(OH)D group status. This product term was then entered into a series of linear and logistic regression models as a predictor variable together with the supplementation group and 25(OH)D group variables. Outcome measurements were internalizing, externalizing, and total problem scores in the linear regression models, and the dichotomous clinically significant problems variables in the logistic regression models. In the linear regression models, *P*-values for the interaction term were 0.22, 0.30, and 0.09 for the three outcome variables, respectively. The corresponding *P*-values for the logistic regression models were 0.59, 0.12, and 0.24, respectively.

An analysis split by sex demonstrated a higher prevalence of total problems (9% vs 2 %), internalizing problems (11% vs 6%), and externalizing problems (15% vs 4%) in boys compared to girls (see **eTables 3** and **4** for participant characteristics divided by sex). To account for potential statistical interaction, a *sex * intervention group* product term was created and entered as the second step in three separate logistic regression models, with dichotomous CBCL internalizing problems, dichotomous CBCL externalizing problems, and dichotomous CBCL total problems, respectively, as the outcome variables. No significant interaction was found for internalizing or externalizing problems (omnibus test of model coefficients was .26, and .52, respectively. For total problems, however, the interaction test was significant (*P* = .02) when comparing a model controlling for supplementation group, and sex, compared to a model also including the interaction term. Neither the analysis with sexes combined, nor the one split by sex, demonstrated a significant association between the supplementation group and the dichotomous total problems outcome variable, however. Given this, as well as the low total number of children with T values above the clinical cut-off point for total problems, all analyses were performed with sexes combined.

© 2023 Sandboge S et al. *JAMA Network Open*.

eReferences

1. The CES-D Scale: A Self-Report Depression Scale for Research in the General Population - Lenore Sawyer Radloff, 1977. Accessed October 9, 2022. https://journals.sagepub.com/doi/abs/10.1177/014662167700100306

2. Blom G. Statistical estimates and transformed beta-variables. Published online 1958. Accessed October 13, 2022. http://urn.kb.se/resolve?urn=urn:nbn:se:su:diva-75457

3. Tuovinen S, Räikkönen K, Holmlund-Suila E, et al. Effect of High-Dose vs Standard-Dose Vitamin D Supplementation on Neurodevelopment of Healthy Term Infants: A Randomized Clinical Trial. *JAMA Netw Open*. 2021;4(9):e2124493. doi:10.1001/jamanetworkopen.2021.24493

4. Holick MF, Binkley NC, Bischoff-Ferrari HA, et al. Evaluation, treatment, and prevention of vitamin D deficiency: an Endocrine Society clinical practice guideline. *J Clin Endocrinol Metab*. 2011;96(7):1911-1930. doi:10.1210/jc.2011-0385

© 2023 Sandboge S et al. *JAMA Network Open*.

eTable 1. Attrition Table for Participants vs Nonparticipants

Characteristic	Non-participants (n = 628)[a]		Participants (n = 346)		MD (95% CI)	*P* value
**Child**						
Female sex, No. (%)	321 (51.1)	628	164 (47.4)	346		.27
Gestational length, mean (SD), d	281.5 (7.7)	628	280,8 (7.5)	346	-0.7 (-1.7; 0.3)	.14
Season of birth		628		346		.05
Winter, No. (%)	132 (21.0)		57 (16.5)			
Spring, No. (%)	271 (43.2)		129 (37.3)			
Summer, No. (%)	129 (20.5)		88 (25.4)			
Autumn, No. (%)	96 (15.3)		72 (20.8)			
Length of breastfeeding, mean (SD), m	*10.4 (5.6)*	*509*	*11.1 (5.6)*	*344*	*0.8 (0.01; 1.5)*	*.05*
Belonging to 1200 IU group, No. (%)	247 (49.5)	499	177 (51.2)	346		.64
**Mother**						
Age at delivery, mean (SD), y	31.0 (4.6)	534	31.4 (4.1)	345	0.3 (-0.3; 0.9)	.28
Smoking at childbirth (yes), No. (%)	*105 (19.5)*	*538*	*43 (12.5)*	*345*		*.01*
Pregnancy 25(OH)D, mean (SD), (ng/mL)	*32.3 (7.8)*	*517*	*33.6 (8.5)*	*291*	*1.2 (0.04; 2.4)*	*.04*
25(OH)D < 30 ng/mL, No. (%)	*208 (40.2)*	*517*	*96 (33.0)*	*291*		*.04*
Educational level (high), No. (%)	*319 (71.2)*	*448*	*245 (83.6)*	*293*		*<.001*
Depressive symptoms at childbirth, median (IQR), score[b]	11 (7)	455	11 (8)	320		.94
CES-D score ≥ 16[c] No. (%)	112 (24.6)	455	73 (22.8)	320		.56
**Father**						
Educational level (high), No. (%)	264 (57.1)	462	178 (66.7)	267		.01

Statistically significant differences in *italics*. Abbreviations: MD, mean difference; CI, confidence interval; SD, standard deviation; d, days, m, months; y, years; 25(OH)D, serum 25 hydroxyvitamin D, IQR, Interquartile range; CES-D, the Center for Epidemiological Studies Scale

[a]An additional 13 children were originally included in the study population, 12 of whom were excluded due to not fulfilling inclusion criteria and 1 who was diagnosed with a rare genetic disorder after inclusion. These children were not included in the attrition analysis.

[b]Depressive symptoms were measured using the Center for Epidemiological Studies Scale.

[c]CES-D of 16 or above reflect at least moderate depressive symptomatology

© 2023 Sandboge S et al. *JAMA Network Open*.

eTable 2. Associations Between Covariates and Child Behavior Checklist Scores[a]

	Internalizing problems			Externalizing problems			Total problems		
	MD	(95% CI)	p	MD	(95% CI)	p	MD	(95% CI)	p
**Child**									
**At birth**									
Sex, female vs. male (ref.)	-0.03	-0.24; 0.19	.80	-0.41	-0.62; -0.20	<.001	-0.36	-0.57; -0.15	<.001
Length of gestation, d	-0.004	-0.02; 0.01	.55	-0.001	-0.02; 0.01	.94	-0.004	-0.02; 0.01	.57
Season of birth, winter (ref.)									
Spring	0.31	-0.01; 0.63	.05	0.12	-0.17; 0.42	.42	0.15	-0.16; 0.46	.33
Summer	0.37	0.02; 0.72	.04	0.25	-0.10; 0.59	.16	0.31	-0.04; 0.66	.08
Autumn	0.36	-0.01; 0.73	.06	0.25	-0.12; 0.62	.18	0.33	-0.02; 0.69	.07
Pregnancy 25(OH)D concentration, ng/mL	-0.004	-0.01; 0.002	.20	0.00	-0.01; 0.01	.89	-0.003	-0.01; 0.002	.29
**At 12-month follow-up**									
Breastfeeding, m	-0.001	-0.02; 0.02	.94	-0.003	-0.02; 0.02	.78	-0.004	-0.02; 0.02	.71
**At follow-up**									
Age, y	0.03	-0.21; 0.27	.79	-0.02	-0.26; 0.22	.86	-0.02	-0.26; 0.22	.88
**Parent**									
Single parent at 6-8 y follow-up, yes vs. no (ref.)	0.17	-0.26; 0.60	.44	-0.03	-0.46; 0.40	.91	0.04	-0.39; 0.47	.85
**Mother**									
Age, y	0.01	-0.02; 0.03	.72	-0.01	-0.03; 0.02	.69	-0.002	-0.03; 0.02	.90
Smoking at childbirth, yes vs no (ref.)	-0.002	-0.34; 0.33	.99	0.22	-0.11; 0.56	.19	0.12	-0.21; 0.46	.47
Depressive symptoms at childbirth, CES-D score[b]	0.17	0.05; 0.29	.01	0.15	0.02; 0.28	.02	0.19	0.06; 0.31	.003
Educational level, low vs high (ref.)	0.23	-0.08; 0.54	.14	0.06	-0.26; 0.37	.71	0.11	-0.20; 0.42	.48
**Father**									
Educational level, low vs high (ref.)	0.08	-0.18; 0.33	.55	0.05	-0.20; 0.31	.67	0.02	-0.23; 0.27	.87

Statistically significant differences in *italics*. Abbreviations: Childhood Behavior Checklist; MD, mean difference; d, days; m, months; y, years; 25(OH)D, serum 25 hydroxyvitamin D; CES-D, Center for Epidemiological Studies Scale.

[a]CBCL raw scores square root transformed due to skewness and converted to Z-scores (0 = mean, 1 = 1 SD).

[b]Depressive symptoms were measured using the Center for Epidemiological Studies Scale, square root transformed due to skewness

© 2023 Sandboge S et al. *JAMA Network Open*.

eTable 3. Study Participant Characteristics by Sex

Characteristic	Female (n = 164)		Male (n = 182)	
**Baseline characteristics**				
**Child**				
Belonging to 1200 IU group, No. (%)	84 (51.2)	164	93 (51.1)	182
Gestational length, mean (SD), d	281.3 (7.4)	164	280.4 (7.7)	182
Season of birth		164		182
Winter, No. (%)	25 (15.2)		32 (17.6)	
Spring, No. (%)	66 (40.2)		63 (34.6)	
Summer, No. (%)	38 (23.2)		50 (27.5)	
Autumn, No. (%)	35 (21.2)		37 (20.3)	
**Mother**				
Age at delivery, mean (SD), y	31.5 (4.2)	164	31.2 (4.1)	181
Smoking at childbirth (yes), No. (%)	15 (9.3)	162	24 (13.4)	179
Pregnancy 25(OH)D, mean (SD), (ng/mL)	33.3 (7.5)	138	33.8 (9.4)	153
25(OH)D < 30 ng/mL, No. (%)	43 (31.2)	138	53 (34.6)	153
Educational level (high), No. (%)	123 (89.1)	138	122 (78.7)	155
Depressive symptoms at childbirth, median (IQR), score[a]	11.0 (6.0)	150	10.0 (7.0)	168
CES-D score ≥ 16 [b] No. (%)	31 (20.7)	150	41 (24.4)	168
**Father**				
Educational level (high), No. (%)	100 (73.5)	136	98 (64.9)	151
**12-month follow-up**				
Length of breastfeeding, mean (SD), m	11.5 (5.5)	162	10.8 (5.7)	182
**6–8-year follow-up**				
**Child**				
Age, mean (SD), y	7.1 (0.4)	164	7.1 (0.4)	182
**Parent**				
Single at follow-up, No. (%)	7 (4.5)	155	16 (11.1)	144

Statistically significant differences in *italics*. Abbreviations: IU, International units; MD, mean difference; CI, confidence interval; SD, standard deviation; d, days, m, months; y, years; 25(OH)D, serum 25 hydroxyvitamin D; IQR, Interquartile range, CES-D, Center for Epidemiological Studies Scale.

[a]Depressive symptoms were measured using the Center for Epidemiological Studies Scale.

[b]CES-D of 16 or above reflect at least moderate depressive symptomatology

© 2023 Sandboge S et al. *JAMA Network Open*.

eTable 4. Child Psychiatric Symptoms and Problems by Sex

	**Female (n = 164)**	**Male (n = 182)**	**MD (95% CI)**	***P* value**
**Clinically significant problems**[a]				
Internalizing problems, No. (%)	10 (6.1)	20 (12.2)		.11
Externalizing problems, No. (%)	*7 (4.3)*	*27 (14.8)*		*<.001*
Total problems, No. (%)	*3 (1.8)*	*16 (8.8)*		*.01*
**CBCL raw scores**				
Internalizing problems, median (IQR)	5.0 (5.0)	4.0 (6.0)		.68
Externalizing problems, median (IQR)	*4.0 (6.0)*	*6.0 (9.0)*		*<.001*
Total problems, median (IQR)	*17.5 (15.8)*	*22.0 (21.0)*		*.002*
**CBCL T scores**				
Internalizing problems T score, mean (SD)	*49.2 (9.0)*	*51.2 (9.2)*	*2.1 (0.2; 4.0)*	*.03*
Externalizing problems T score, mean (SD)	*49.6 (7.9)*	*52.4 (9.5)*	*2.8 (0.9; 4.7)*	*.003*
Total problems T score, mean (SD)	*48.1 (8.2)*	*51.0 (9.0)*	*2.9 (1.1; 4.7)*	*.002*

Statistically significant differences in *italics*. Abbreviations: MD, mean difference; CI, confidence interval; IQR, interquartile range; SD, standard deviation; CBCL, Childhood Behavior Checklist
P values for proportions calculated using χ^2 test; *P* values for medians calculated using Mann-Whitney U test; *P* values for means calculated using T-test, two-sided *P* values, equal variance assumed
[a]CBCL raw scores converted to T scores and dichotomized at 64. A T score of 64 or above is considered to reflect clinically significant problems.

© 2023 Sandboge S et al. *JAMA Network Open*.

eTable 5. Associations Between Vitamin D_3 Concentrations at Ages 1 and 2 y and Psychiatric Symptoms at Ages 6 to 8 y[a]

		Internalizing problems			Externalizing problems			Total problems		
**Association with CBCL scores**										
	**n**	**MD**	**(95% CI)**	***P***	**MD**	**(95% CI)**	***P***	**MD**	**(95% CI)**	***P***
12m. 25(OH)D concentration, ng/mL										
Model 1[b]	324	*-0.01*	*-0.02; -0.001*	*.04*	-0.01	-0.01; 0.01	.33	-0.01	-0.01; 0.003	.16
Model 2[c]	300	-0.01	-0.02; 0.004	.26	-0.002	-0.01; 0.01	.69	-0.003	-0.01; 0.01	.57
24m. 25(OH)D concentration, ng/mL										
Model 1	343	*-0.01*	*-0.02; -0.002*	*.02*	-0.01	-0.02; 0.003	.22	-0.01	-0.02; 0.001	.08
Model 2	315	*-0.01*	*-0.02; -0.002*	*.02*	-0.01	-0.02; 0.004	.24	-0.02	-0.02; 0.001	.09
**Association with Clinically significant problems**										
	**n**	**OR**	**(95% CI)**	***P***	**OR**	**(95% CI)**	***P***	**OR**	**(95% CI)**	***P***
12m. 25(OH)D concentration, ng/mL										
Model 1	324	*0.93*	*0.90; 0.97*	*0.001*	0.98	0.95; 1.02	0.32	0.99	0.95; 1.03	0.49
Model 2	300	*0.94*	*0.89; 0.98*	*0.01*	0.98	0.94; 1.02	0.27	1.00	0.95; 1.04	0.91
24m. 25(OH)D concentration, ng/mL										
Model 1	343	*0.95*	*0.91; 0.98*	*0.01*	0.97	0,94; 1.01	0.12	0.96	0.92; 1.01	0.10
Model 2	315	*0.95*	*0.91; 0.99*	*0.01*	0.97	0.94; 1.01	0.12	0.97	0.93; 1.02	0.26

Statistically significant differences in italics. Abbreviations: 25(OH)D, serum 25 hydroxyvitamin D; CBCL, Childhood Behavior Checklist; MD, mean difference; CI, confidence interval; m, months; OR, odds ratio.

[a]Psychiatric symptoms assessed using the Childhood Behavior Checklist. In linear models, raw scores were square root transformed due to skewness and converted to Z-scores (0 = mean, 1 = 1 SD). In logistic models, scores were converted to T scores and dichotomized at 64 or above to reflect clinically significant problems. ORs and 95% CIs from logistic regression analyses show odds of belonging to a group with clinically significant problems per unit increase of 25(OH)D concentration.

[b]Unadjusted model

[c]Adjusts for sex, maternal depressive symptoms at birth, the season of 25(OH)D measurement, and parental single status at follow-up.

© 2023 Sandboge S et al. *JAMA Network Open*.

eTable 6. Prevalence of Child Behavior Checklist *T* Scores Within the Clinical Range for Maternal Vitamin D_3 and Supplementation Status Subgroups[a]

	**25(OH)D< 30 / 400 IU group (n = 49)**	**25(OH)D> 30 / 400 IU group (n = 94)**	*P* value	**25(OH)D <30 / 1200 IU group (n = 47)**	*P* value	**25(OH)D> 30 / 1200 IU group (n = 101)**	*P* value
**Clinically significant problems**							
Internalizing problems, No. (%)	9 (18.4), (Ref.)	9 (9.6)	.22	3 (6.4)	.09	5 (5.0)	.01
Externalizing problems, No. (%)	6 (12.2), (Ref.)	6 (6.4)	.24	3 (6.4)	.33	12 (11.9)	.95
Total problems, No. (%)	5 (10.2), (Ref.)	4 (4.3)	.18	2 (4.3)	.28	6 (5.9)	.35

[a]Psychiatric symptoms assessed using the Childhood Behavior Checklist. Raw scores were converted to T scores and dichotomized at 64 or above to reflect clinically significant problems.

© 2023 Sandboge S et al. *JAMA Network Open*.

eTable 7. Association of Subgroups by Vitamin D_3 Supplementation and Maternal Vitamin D_3 Level With Psychiatric Symptoms at Ages 6 to 8 y[a]

In the reference group are children born to mothers with higher vitamin D levels during pregnancy (25(OH)D ≥30 ng/mL) who were subsequently randomized into 1200 IU group (n=101-91).

	**Maternal 25(OH)D <30 ng/mL**				**Maternal 25(OH)D ≥30 ng/mL**	
	**400 IU group (n=49–48)**		**1200 IU group (n=47–44)**		**400 IU group (n=94-83)**	
	**MD or OR (95% CI)**	***P* value**	**MD or OR (95% CI)**	***P* value**	**MD or OR (95% CI)**	***P* value**
**Internalizing problems**						
Score						
Model 1[b]	0.41 (0.07 to 0.75)	.02	0.09 (-0.20 to 0.37)	.56	0.01 (-0.33 to 0.36)	.94
Model 2[c, d]	0.37 (0.03 to 0.72)	.04	0.07 (-0.22 to 0.36)	.63	-0.12 (-0.48 to 0.23)	.50
**Clinically significant problems**						
Model 1	4.32 (1.36 to 13.70)	.01	2.03 (0.66 to 6.30)	.22	1.31 (0.30 to 5.72)	.72
Model 2	4.75 (1.28 to 17.56)	.02	2.39 (0.67 to 8.51)	.18	1.45 (0.29 to 7.25)	.65
**Externalizing problems**						
Score						
Model 1	0.07 (-0.28 to 0.41)	.71	-0.14 (-0.43 to 0.14)	.32	-0.06 (-0.41 to 0.30)	.76
Model 2	0.02 (-0.34 to 0.38)	.91	-0.12 (-0.42 to 0.17)	.41	-0.10 (-0.47 to 0.26)	.58
**Clinically significant problems**						
Model 1	1.04 (0.36 to 2.94)	.95	0.51 (0.18 to 1.41)	.19	0.51 (0.14 to 1.89)	.31
Model 2	0.98 (0.31 to 3.10)	.98	0.59 (0.20 to 1.75)	.34	0.28 (0.05 to 1.41)	.12
**Total problems**						
Score						
Model 1	0.31 (-0.03 to 0.65)	.08	-0.10 (-0.38 to 0.18)	.48	-0.01 (-0.36 to 0.33)	.94
Model 2	0.23 (-0.12 to 0.58)	.19	-0.07 (-0.36 to 0.22)	.63	-0.11 (-0.47 to 0.25)	.55
**Clinically significant problems**						
Model 1	1.80 (0.52 to 6.21)	.35	0.70 (0.19 to 2.58)	.60	0.70 (0.14 to 3.63)	.67
Model 2	1.89 (0.47 to 7.61)	.37	0.92 (0.23 to 3.63)	.90	0.36 (0.04 to 3.38)	.37

Abbreviations: IU, International units; MD,mean difference; OR, odds ratio; CI, confidence interval; SD, standard deviation; CBCL, Childhood Behavior Checklist

[a]Psychiatric symptoms assessed using the Childhood Behavior Checklist. In linear models, raw scores were square root transformed due to skewness and converted to Z-scores (0 = mean, 1 = 1

© 2023 Sandboge S et al. *JAMA Network Open*.

SD). Mean differences were calculated compared to the children in the 400 IU supplementation group whose mothers had 25(OH)D levels during pregnancy < 30 ng/mL. In logistic regression models, raw scores were converted to T-scores and dichotomized at 64 or above to reflect clinically significant problems. ORs and 95% CIs from logistic regression analyses show odds of belonging to a group with clinically significant problems for each sub-group compared to the same reference group as above.
[b]Unadjusted model
[c]Adjusts for sex, season of birth, maternal depressive symptoms at birth, and parental single status at follow-up.
[d]In the adjusted models, n for the 25(OH)D< 30 / 400 IU, 25(OH)D≥ 30 / 400 IU, 25(OH)D <30 / 1200 IU, and 25(OH)D≥ 30 / 1200 IU groups were 48, 83, 44, and 91, respectively.

© 2023 Sandboge S et al. *JAMA Network Open*.

eFigure. Distribution of Raw Internalizing, Externalizing, and Total Problems Sum Scores by Supplementation Group

eFigure 1a. Distribution of internalizing problems sum scores by supplementation group

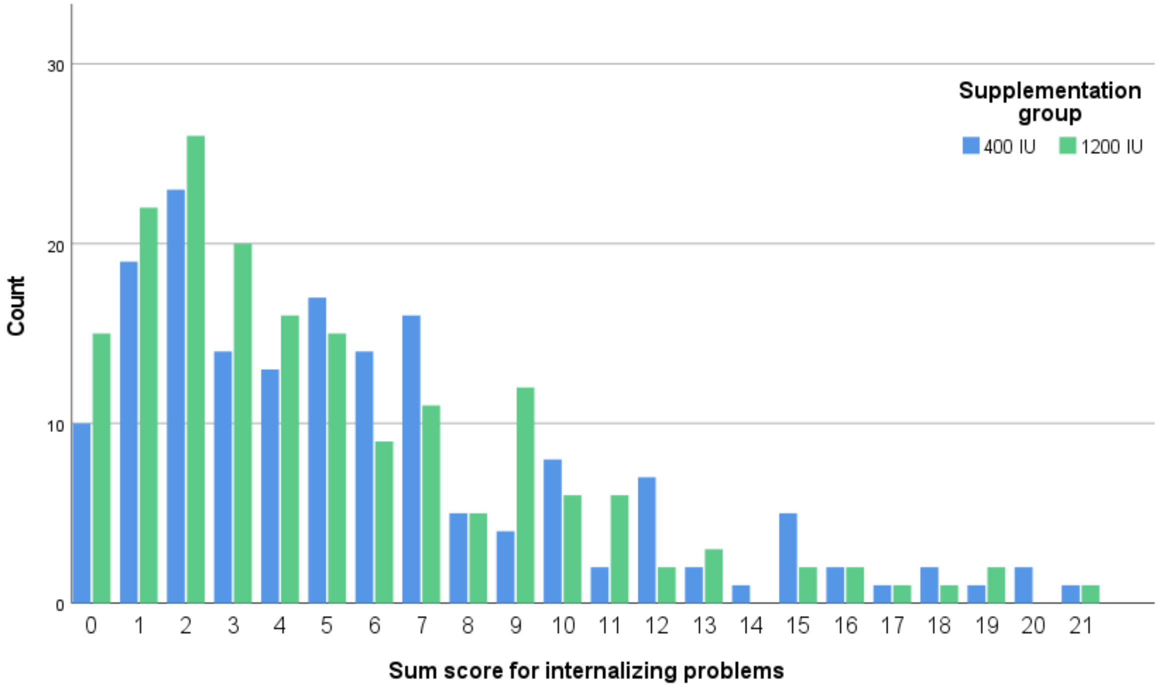

eFigure 1b. Distribution of externalizing problems sum scores by supplementation group

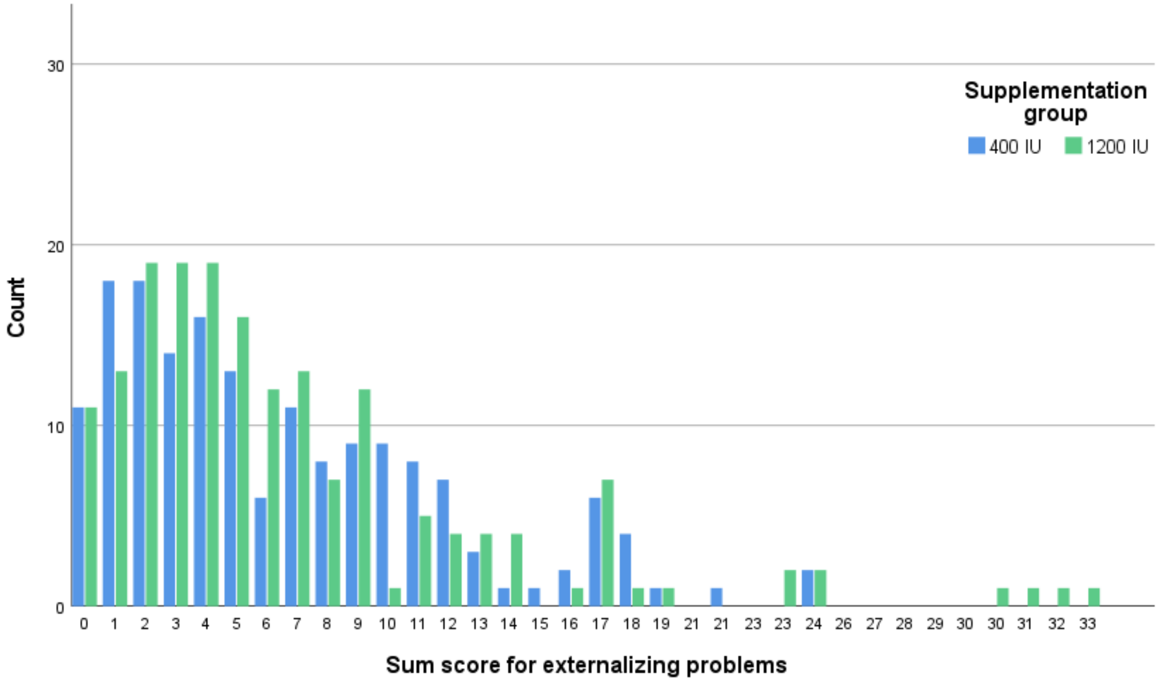

© 2023 Sandboge S et al. *JAMA Network Open*.

eFigure 1c. Distribution of total problems sum scores by supplementation group

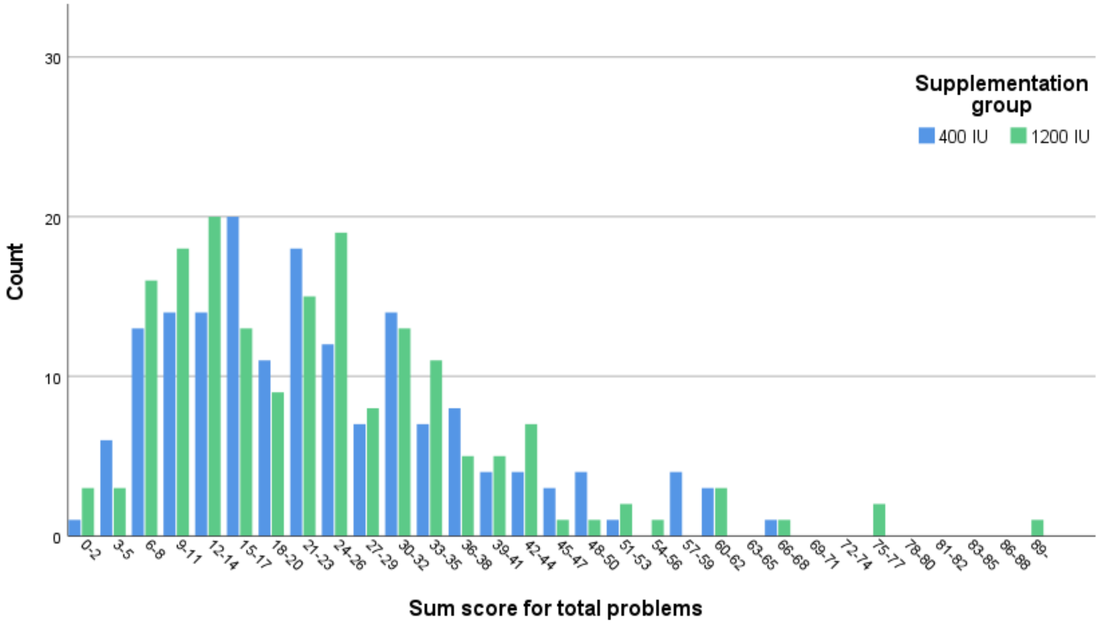

© 2023 Sandboge S et al. *JAMA Network Open*.
