# Supplementary material for: Effect of Vitamin D3 Supplementation in the First 2 Years of Life on Psychiatric Symptoms at Ages 6 to 8 Years: A Randomized Clinical Trial
Source: JAMA Netw Open. 2023 May 19;6(5):e2314319. doi: 10.1001/jamanetworkopen.2023.14319 (PMC10199342; doi:10.1001/jamanetworkopen.2023.14319)
Supplement: Supplement 3. — Data Sharing Statement [file jamanetwopen-e2314319-s003.pdf]

**Data Sharing Statement**

Sandboge. Effect of Vitamin $D_3$ Supplementation in the First 2 Years of Life on Psychiatric Symptoms at Ages 6 to 8 Years: A Randomized Clinical Trial. *JAMA Netw Open*. Published online May 19, 2023. doi:10.1001/jamanetworkopen.2023.14319

**Data**
**Data available:** No

© 2023 Sandboge S et al. *JAMA Network Open*.
